# *Bifidobacterium breve* UCC2003 Induces a Distinct Global Transcriptomic Program in Neonatal Murine Intestinal Epithelial Cells

**DOI:** 10.1016/j.isci.2020.101336

**Published:** 2020-07-02

**Authors:** Raymond Kiu, Agatha Treveil, Lukas C. Harnisch, Shabhonam Caim, Charlotte Leclaire, Douwe van Sinderen, Tamas Korcsmaros, Lindsay J. Hall

**Affiliations:** 1 Gut Microbes & Health, Quadram Institute Bioscience, Norwich Research Park, Norwich NR4 7UQ, UK; 2 Earlham Institute, Norwich Research Park, Norwich NR4 7UZ, UK; 3 APC Microbiome Ireland & School of Microbiology, University College Cork, Cork T12YT20, Ireland; 4 Norwich Medical School, University of East Anglia, Norwich Research Park, Norwich NR4 7TJ, UK; 5 Chair of Intestinal Microbiome, School of Life Sciences, Technical University of Munich, 85354 Freising, Germany; 6 ZIEL – Institute for Food & Health, Technical University of Munich, 85354 Freising, Germany

**Keywords:** Microbiology, Microbiome, Transcriptomics

## Abstract

The underlying health-driving mechanisms of *Bifidobacterium* during early life are not well understood, particularly how this microbiota member may modulate the intestinal barrier via programming of intestinal epithelial cells (IECs). We investigated the impact of *Bifidobacterium breve* UCC2003 on the transcriptome of neonatal murine IECs. Small IECs from two-week-old neonatal mice administered *B*. *breve* UCC2003 or PBS (control) were subjected to global RNA sequencing, and differentially expressed genes, pathways, and affected cell types were determined. We observed extensive regulation of the IEC transcriptome with ∼4,000 genes significantly up-regulated, including key genes linked with epithelial barrier function. Enrichment of cell differentiation pathways was observed, along with an overrepresentation of stem cell marker genes, indicating an increase in the regenerative potential of the epithelial layer. In conclusion, *B*. *breve* UCC2003 plays a central role in driving intestinal epithelium homeostatic development during early life and suggests future avenues for next-stage clinical studies.

## Highlights

• *B. breve* administration significantly alters the murine neonatal IEC transcriptome
• Genes/pathways involved in epithelial barrier function are particularly impacted
• *Bifidobacterium* may target the IEC stem cell compartment to induce regeneration

## Graphical Abstract

**Figure:**
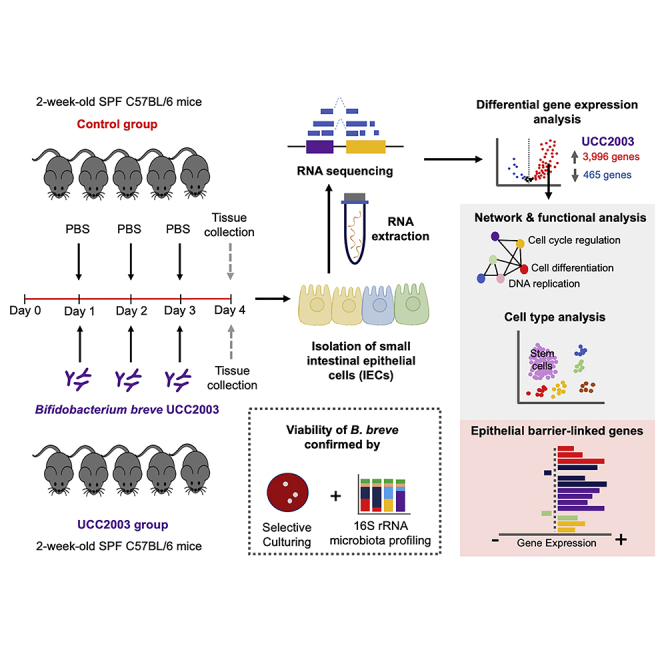

## Introduction

*Bifidobacterium* represents a keystone member of the early life gut microbiota (Arrieta et al., 2014; O'Neill et al., 2017; Derrien et al., 2019). Certain species and strains are found at high levels in vaginally delivered breast-fed infants including *Bifidobacterium longum* subsp. *infantis*, *B*. *longum* subsp. *longum*, *Bifidobacterium bifidum*, *Bifidobacterium pseudocatenulatum,* and *Bifidobacterium breve* (Dominguez-Bello et al., 2010; Mikami et al., 2012; Nagpal et al., 2017; Stewart et al., 2018). As a dominant member of the neonatal gut microbiota, *Bifidobacterium* is associated with metabolism of breast milk, modulation of host immune responses, and protection against infectious diseases (Fukuda et al., 2012; Ling et al., 2016; Robertson et al., 2020; Lawson et al., 2020; Patole et al., 2016; Baucells et al., 2016; Jacobs et al., 2013; Plummer et al., 2018). However, the mechanisms driving improved health outcomes during early life are largely underexplored and are likely strain dependent.

A key interface between *Bifidobacterium* and the host is the intestinal epithelial cell (IEC) barrier (Thoo et al., 2019; Groschwitz and Hogan, 2009). Previous studies have indicated that certain strains of *Bifidobacterium* specifically modulate IEC responses during inflammatory insults, which may help protect from certain gut disorders (Hsieh et al., 2015; Srutkova et al., 2015; Grimm et al., 2015). In murine experimental models, previous work by our group has shown that infant-associated *B*. *breve* UCC2003 modulates cell death-related signaling molecules, which in turn reduces the number of apoptotic IECs (Hughes et al., 2017). This protection from pathological IEC shedding appeared to be via the *B*. *breve* exopolysaccharide (EPS) capsule and the host-immune adaptor protein MyD88. Another strain of *B*. *breve*, NumRes 204 (commercial strain) has also been shown to up-regulate the tight junction (TJ) proteins Claudin 4 and Occludin in a mouse colitis model (Zheng et al., 2014; Plantinga et al., 2011).

Many of the studies to date have focused on the role of *Bifidobacterium* and modulation of IECs in the context of acute or chronic gut inflammation, with expression profiling limited to specific immune or apoptosis signaling targets (Plaza-Diaz et al., 2014, Riedel et al., 2006; Liu et al., 2010; Hsieh et al., 2015). As many of these studies have involved pre-colonization of the gut with *Bifidobacterium* strains, followed by inflammatory insult, this suggests that initial priming during normal “healthy” conditions may modulate subsequent protective responses. Furthermore, these studies have often been performed in adult mice rather than exploring effects during the early life developmental window, where *Bifidobacterium* effects are expected to be most pronounced. Previous work has indicated that there is significant modulation of the neonatal IEC transcriptome in response to gut microbiota colonization, but to date no studies have probed how particular early life-associated microbiota members, like *Bifidobacterium,* may modulate neonatal IEC responses (Pan et al., 2018). Thus, to understand if and how *Bifidobacterium* may modulate IEC homeostasis during the early life developmental window, we administered *B*. *breve* UCC2003 to neonatal mice and profiled transcriptional responses in isolated small intestine IECs using global RNA sequencing (RNA-seq). Our analysis indicated whole-scale changes in the transcriptional program of IECs (∼4,000 significantly up-regulated genes) that appear to be linked to cell differentiation/proliferation and immune development. Notably the stem cell compartment of IECs seemed to elicit the strongest gene signature. These data highlight the role of *B*. *breve* UCC2003 in driving early life epithelial cell differentiation and maturation, impacting intestinal integrity and immune functions, which provides a mechanistic basis for understanding associated health-promoting effects.

## Results

**Figure 1:**
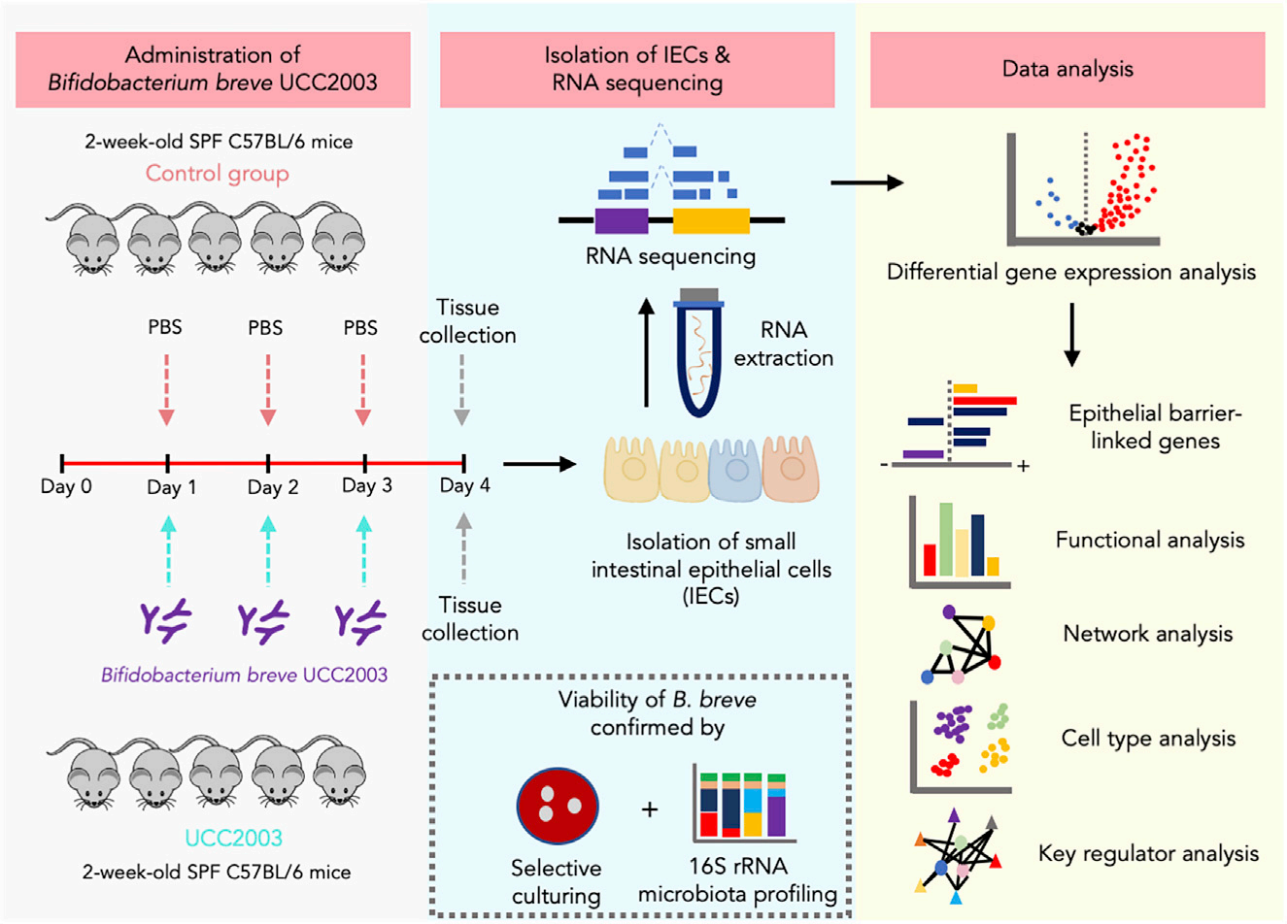
Schematic Representation of the Study Design and *In Silico* Analysis Workflow

To examine the effects of *B*. *breve* UCC2003 on the transcriptional profiles of host IECs under homeostatic conditions, we extracted RNA from isolated IECs of healthy 2-week-old neonatal mice (control group) and mice gavaged with *B*. *breve* UCC2003 for three consecutive days (*n* = 5 per group). Isolated RNAs from IECs were subjected to RNA-seq to determine global mRNA expression (Figure 1). Subsequently, Differential Gene Expression analysis was performed to understand *B*. *breve*-associated gene regulation.

### Minimal Impact of *B*. *breve* UCC2003 on the Wider Neonatal Gut Microbiota

**Figure 2:**
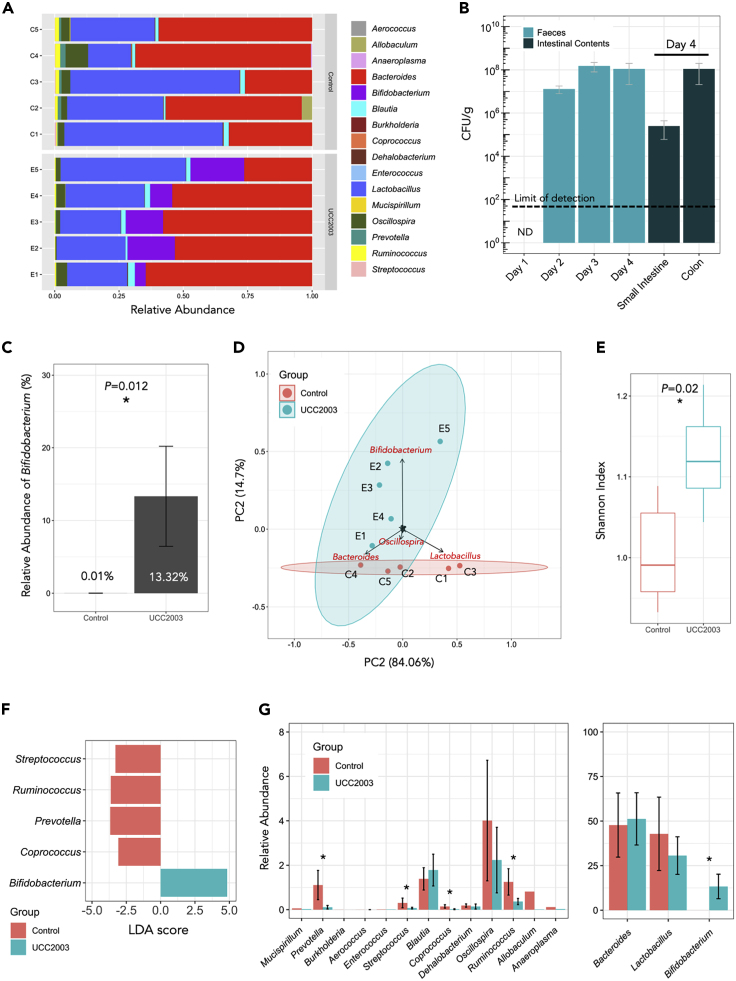
16S rRNA Amplicon Sequencing Analysis of Murine Intestinal Microbiota (A) Genus-level 16S rRNA gene profiling of mice gut microbiota on day 4 (control versus UCC2003). (B) Dynamics of *B*. *breve* UCC2003 load (CFU/g) from day 1 (before *B*. *breve* administration) through day 4. *B. breve* was present in intestines throughout (small intestines and colon; on day 4). ND, non-detectable. Data are represented as mean ± SD. (C) Relative abundance of genus *Bifidobacterium* in UCC2003 group is significantly increased. (D) Principal-component analysis on mice gut microbiota (control versus UCC2003 based on genus-level metataxonomics). (E) Shannon diversity index on mice gut microbiota (control versus UCC2003). Data are represented as mean ± SD. Significance test: t test (∗p < 0.05; two-sided). (F) Linear Discriminant Analysis (LDA) showing enriched taxa in each group (control versus UCC2003). (G) Relative abundance comparison of all genera. ∗p < 0.05 (LDA).

Initially, we examined for the presence of *B*. *breve* UCC2003 in the gut microbiome and impact on the wider microbiota using culture and 16S rRNA microbiota profiling approaches (Figures 2A and 2B). We observed high levels of *B*. *breve* UCC2003 across the 4 days in fecal samples, with higher levels of viable *B*. *breve* UCC2003 within the colon (∼10^8^ CFU/g [colony-forming unit]), when compared with the small intestine (∼10^5^ CFU/g; Figure 2B). Based on 16S rRNA analysis, relative abundance of *Bifidobacterium* increased significantly in the UCC2003 group (p = 0.012) following bacterial administration, whereas the control group displayed very low relative *Bifidobacterium* abundance (∼0.01%; Figure 2C). Principal-component analysis (PCA) on gut microbiota profiles (control versus UCC2003) showed a distinct change in microbial community composition in the UCC2003 group primarily driven by increased relative abundance of *Bifidobacterium*, which may also correlate with increased overall microbial diversity in the UCC2003 group (Figures 2D and 2E). Linear Discriminant Analysis also indicated that *Bifidobacterium* was uniquely enriched in UCC2003 group, and that microbiota members with low relative abundance (<2%) such as *Streptococcus*, *Ruminococcus*, *Prevotella,* and *Coprococcus* were significantly lower (Figures 2F and 2G). Overall, administration of *B*. *breve* UCC2003 appeared to minimally impact the wider gut microbiota, without significantly altering relative abundance of other major resident taxa including *Lactobacillus*, *Bacteroides,* and *Blautia* compared with the control group.

### Impact of *B*. *breve* UCC2003 on the Neonatal Intestinal Epithelial Transcriptome

**Figure 3:**
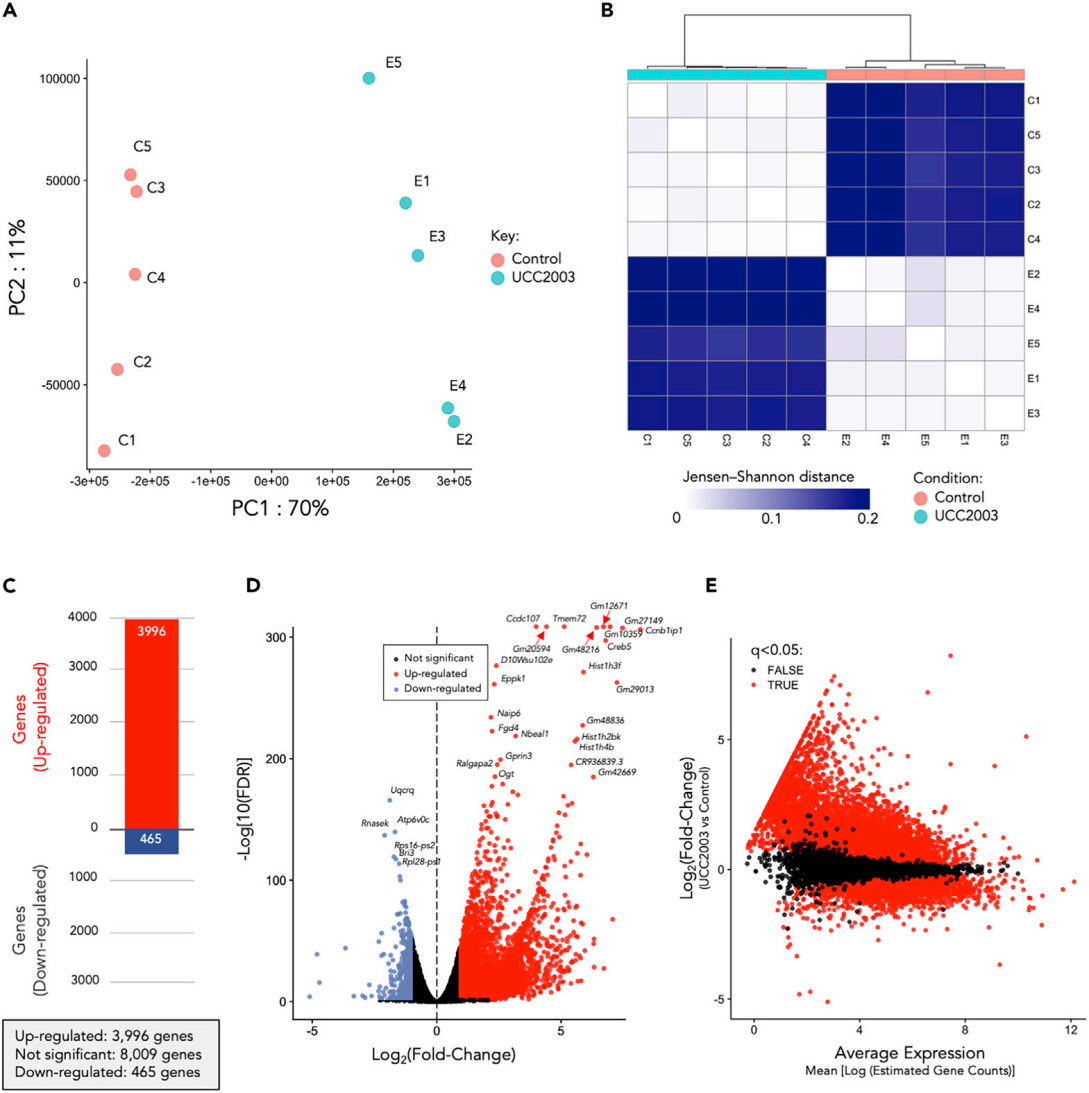
RNA-Seq Analysis and Statistics (A) Principal-component analysis showing distinct overall gene expression profiles across all individual samples based on 12,965 highly expressed genes. See also Table S1. (B) Clustering of individual RNA-seq samples based on Jensen-Shannon distance. Distinct gene expression profiles were demonstrated between these two groups of samples (control versus UCC2003). (C) Total number of differentially expressed genes (DEGs) in UCC2003 group. (D) Volcano plot of global gene expression. Up-regulated DEGs are labeled as red dots, whereas down-regulated DEGs are labeled in blue. (E) MA plot of global gene expression (plot of log-intensity ratios [M-values] versus log-intensity averages [A-values]).

**Figure 4:**
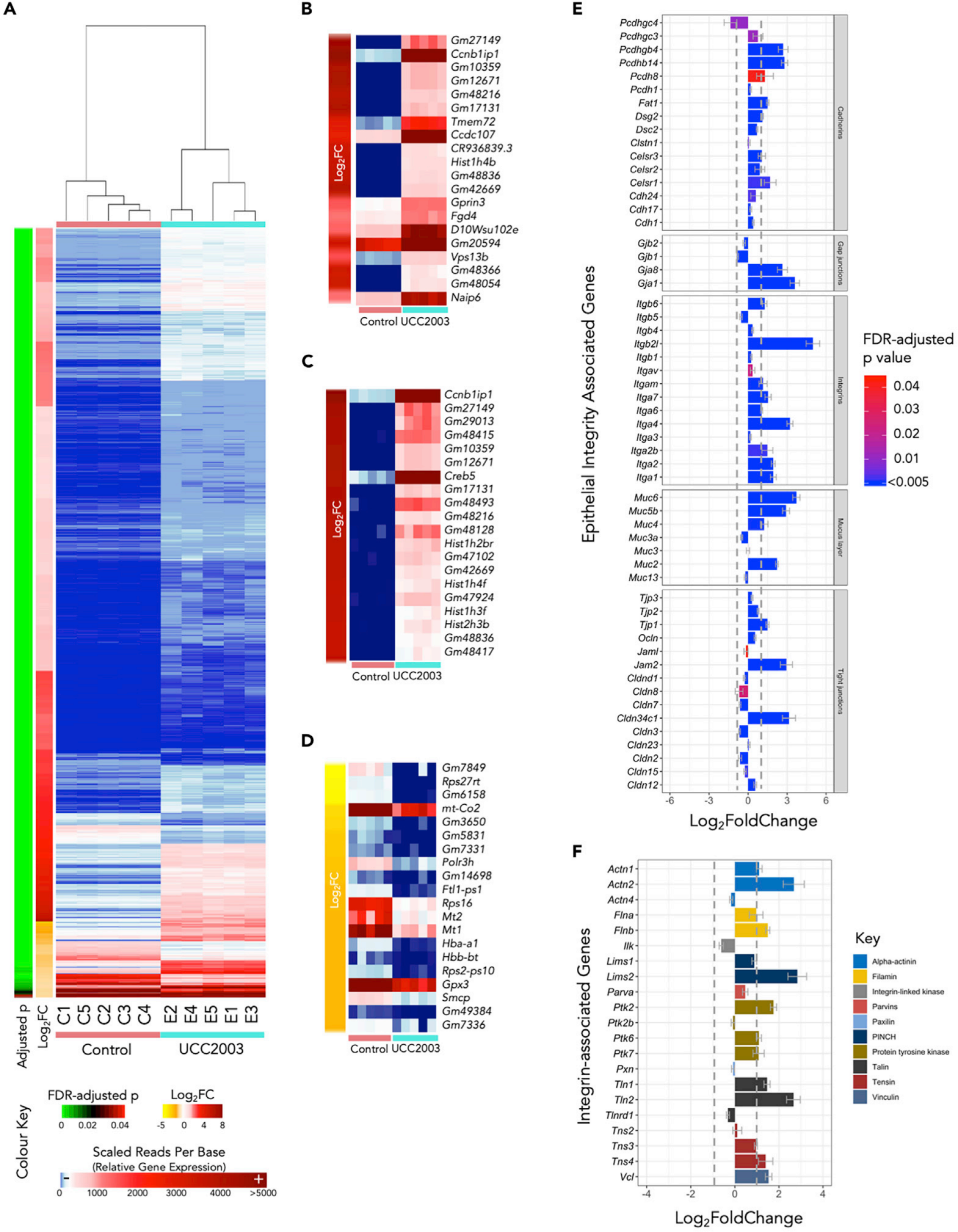
Gene Expression Analysis (A) Heatmap comparison of gene expression profiles of 4,461 DEGs (control versus UCC2003). See also Table S2. (B) Top 20 DEGs ranked by false discovery rate -adjusted p values (q values). (C) Top 20 up-regulated DEGs ranked by log_2_FC (fold change) values. (D) Top 20 down-regulated DEGs ranked by log_2_FC values. (E) Expression of epithelial integrity associated genes in UCC2003 group (q < 0.05). (F) Expression of integrin-associated genes in UCC2003 group. Gray dotted lines in the bar charts indicate the threshold of absolute log_2_FC > 1.0. Data are represented as mean ± SE.

To understand the distribution of samples based on IEC gene expression profiles we performed PCA analysis (Figure 3A; Table S1). All samples clustered according to group (control versus UCC2003), suggesting a significant impact of *B*. *breve* UCC2003 on gene expression profiles, with distance-wise clustering (Jensen-Shannon) also supporting separation of experimental groups (Figure 3B). To define differentially expressed genes (DEGs), we employed a filter of absolute log_2_fold change (LFC) > 1.0 (with adjusted p < 0.05), which equates to a minimum 2-fold change in gene expression (Figures 3C–3E; Table S2). After analysis, a total of 3,996 DEGs were significantly up-regulated, whereas 465 genes were significantly down-regulated in *B*. *breve* UCC2003-supplemented animals when compared with controls (Figures 3C and 4A). Notably, we also performed the same experimental protocol on healthy mice aged 10–12 weeks and did not observe any significant DEGs, suggesting that *B*. *breve* UCC2003 modulation of IECs is strongest within the early life window under homeostatic conditions.

To determine the functional role of DEGs, we examined the most significantly regulated genes ranked by false discovery rate-adjusted p values (or, *q* values). We first looked at the top 20 up-regulated DEGs in the *B*. *breve* UCC2003 experimental group (Figure 4B). Most genes annotated with known biological processes had cell differentiation and cell component organization functions including *Ccnb1ip1*, *Hist1h4b*, *Vps13b,* and *Fgd4* (annotated in the PANTHER Gene Ontology [GO] Slim resource). Two genes were involved in cell death and immune system processes, namely, *Naip6* and *Gm20594* (Table S3). When we ranked the top-regulated genes using LFC, we observed increased expression of *Creb5*, which is involved in the regulation of neuropeptide transcription (cAMP response element-binding protein; CREB) (Figure 4C). CREB is also known to regulate circadian rhythm, and we also identified additional circadian-clock-related genes that were significantly up-regulated including *Per2* and *Per3*. We noted that several top down-regulated DEGs were annotated as genes involved in metal binding, or metal-related genes including *Mt1*, *Mt2*, *Hba-a1*, *Hbb-bt,* and *Ftl1-ps1* (Figure 4D; Table S4).

### Regulation of Intestinal Epithelial Barrier-Associated Genes

As *B*. *breve* strains have been previously shown to modulate certain TJ/barrier-related proteins, we next investigated DEGs associated with intestinal epithelial barrier development/intestinal structural organization (Figure 4E). Several TJ structural-associated DEGs were observed, including Claudin-encoding gene *Cldn34c1* (LFC 3.14), Junction Adhesion Molecules-encoding genes *Jam2* (LFC 2.9), and TJ protein (also called Zonula Occludens protein; ZO)-encoding gene *Tjp1* (LFC 1.49). Genes that encode integrins (involved in regulation of intracellular cytoskeleton) also exhibited a trend of increased expression (13/14; 92.8%). Both Piezo genes, which assist in TJ organization, *Piezo1* (LFC 1.25) and *Piezo2* (LFC 1.9), were significantly up-regulated in the *B*. *breve* UCC2003-treated group.

Over 90% cadherins, proteins associated with the assembly of adherens junctions (Figure 4E), were up-regulated, including *Pcdhb14* (LFC 2.8), *Pcdhgb4* (LFC 2.7), *Pcdh8* (LFC 1.3), *Fat1* (LFC 1.5), and *Dsg2* (LFC 1.1). Interestingly, several genes (4/7; 57.1%) involved in mucous layer generation were significantly up-regulated in the UCC2003 experimental group, including *Muc2* (LFC 2.2), *Muc6* (LFC 3.7), *Muc5b* (LFC 2.9), and *Muc4* (LFC 1.24). Genes *Gja1* (LFC 3.59) and *Gjb8* (LFC 2.63) that encode gap junction proteins were also up-regulated. In addition, we also investigated the differential expression of genes associated with integrin assembly and downstream integrin signaling pathways (Figure 4F). Over 70% (16/21) of these genes were up-regulated, with 52.3% (11/21) significantly increased in gene expression in the UCC2003 group (LFC >1.0).

We observed increased expression of genes associated with IEC barrier development including cadherins, gap junctions, integrins, mucous layer-associated genes, and several key TJ proteins. These strongly induced gene expression profiles suggest that *B*. *breve* UCC2003 is involved in enhancing epithelial barrier development in neonates.

### Modulation of Cell Maturation Processes

We next sought to understand the biological functions of up-regulated DEGs by employing PANTHER GO-Slim functional assignment and process/pathway enrichment analysis (see Figure S1; Tables S5 and S6). DEGs were predominantly involved in general biological processes including cellular process (901 genes) and metabolic process (597 genes; Table S7). At the molecular function level, DEGs were primarily assigned to binding (868 genes) and catalytic activity (671 genes; Table S8), with Olfactory Signaling Pathway and Cell Cycle (biological) pathways also found to be enriched (Table S9).

**Figure 5:**
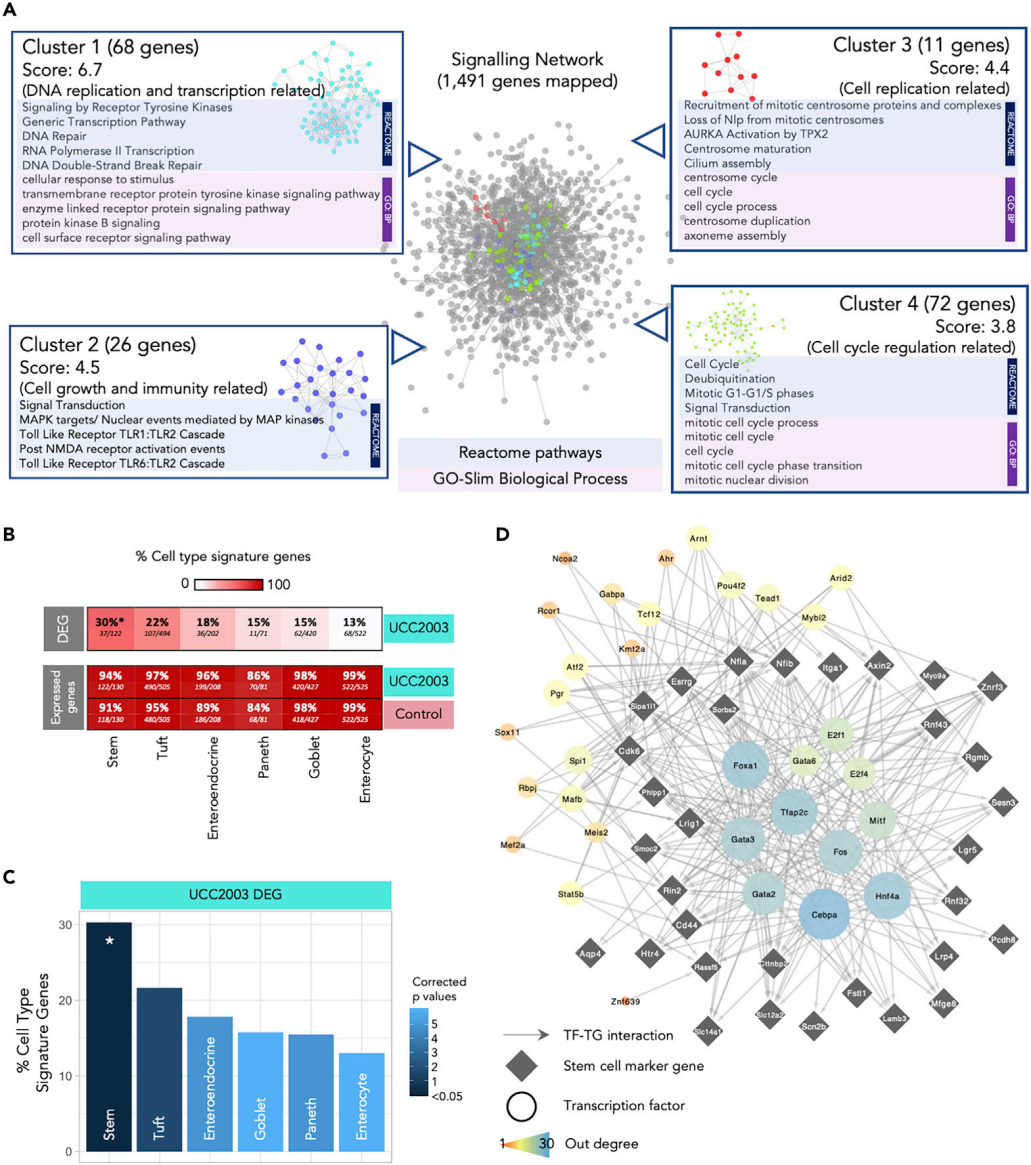
Signaling Network Analysis, IEC Subtyping, and Key Regulator Analysis (A) Cluster analysis of signaling network for significantly up-regulated genes (*n* = 3,996). Representative enriched pathways (Reactome) and GO terms (Biological Process) identified in each individual cluster were listed alongside. See also Table S10. (B) Heat plot showing percentage of cell type signature genes in DEG and expressed genes (both control and UCC2003 groups). All expressed genes are well represented in IEC cell type signature genes. (C) Cell type analysis on IEC DEGs using known cell-specific signature genes. Stem cells were statistically over-represented in DEGs. ∗p < 0.05. See also Table S11. (D) Key regulators of stem cell DEGs.

To delve further into the data, we constructed a signaling network based on up-regulated DEGs (*n* = 3,996) with the aim of identifying specific gene networks involved in important signaling pathways (Figure 5A). Overall, 1,491 DEGs were successfully mapped (37.3%) to a signaling network that comprised 8,180 genes. Four individual clusters of genes were detected, with functional assignment and pathway analysis implemented on these clusters (Figure 5A). All gene clusters were associated with cell differentiation and maturation, with cluster 1 (68 genes) linked specifically with DNA replication and transcription, cluster 2 (26 genes) with cell growth and immunity, cluster 3 (11 genes) with cell replication, and cluster 4 (72 genes) related to cell cycle and cell division (Table S10).

### Intestinal Cell Type Analysis on DEGs Identifies Significant Enrichment of Epithelial Stem Cells

IECs include several absorptive and secretory cell types, namely, enterocytes, Paneth cells, goblet cells, enteroendocrine cells, tuft cells, and stem cells. As these cells perform different functions in the gut, it was important to understand whether *B*. *breve* UCC2003 had a cell type-specific effect on the intestinal epithelium. Using known cell type-specific gene markers (Haber et al., 2017), we identified cell type gene signatures modulated within the UCC2003 group (Figures 5B and 5C). Importantly, all cell type markers were well represented in the expressed genes of the whole IEC transcriptomics data from both groups (control + UCC2003), thus validating the presence of all IEC types in our study data (Figure 5B). Cell type analysis of genes differentially expressed after *B*. *breve* UCC2003 supplementation revealed that stem cell marker genes were significantly enriched (30%; p < 0.05) among the six IEC types (Table S11). Signatures of other cell types were also present (linking to marker genes in the DEG list) but not significantly overrepresented: tuft cells (22%), enteroendocrine cells (18%), goblet cells (15%), Paneth cells (15%), and enterocytes (13%; Figure 5C). These data indicated that intestinal epithelial stem cells, cells primarily involved in cell differentiation, were the primary cell type whose numbers and transcriptomic program were regulated by *B*. *breve* UCC2003.

Further investigation of this stem cell signature revealed that of the 37 differentially expressed marker genes, 35 are up-regulated in the presence of *B*. *breve* UCC2003. This indicates an increase in the quantity of stem cells or semi-differentiated cells in the epithelium, consistent with the overrepresentation of cell cycle- and DNA replication-associated genes observed in the whole differential expression dataset. Functional analysis of the 37 stem cell signature genes revealed only one overrepresented process—Regulation of Frizzled by ubiquitination (p < 0.05), which is a subprocess of WNT signaling. WNT signaling is important in maintaining the undifferentiated state of stem cells (Nusse, 2008).

Finally, we employed a network approach to predict key transcription factor (TF) regulators of the differentially expressed stem cell marker genes, through which *B*. *breve* UCC2003 may be acting (Figure 5D). Using the TF-target gene database, DoRothEA, we identified expressed TFs known to regulate these genes (Garcia-Alonso et al., 2019; Holland et al., 2019). Five genes had no known and expressed regulator, and thus were excluded. Hypergeometric significance testing was used to identify which of these TFs are the most influential (see Methods for details). This analysis identified 32 TF regulators (Figure 5D). Of these regulators, 12 were differentially expressed in the IEC dataset (all up-regulated): *Fos*, *Gabpa*, *Rcor1*, *Arid2*, *Tead1*, *Mybl2*, *Mef2a*, *Ahr*, *Pgr*, *Kmt2a*, *Ncoa2,* and *Tcf12*. Functional analysis of all the TF regulators and their targeted genes together, revealed overrepresented functions relating to WNT signaling, histone methylation for self-renewal and proliferation of hematopoietic stem cells, and nuclear receptor (incl. estrogen) signaling (Table S12). These data indicate that *B*. *breve* UCC2003 directly affects key transcriptomic programs that regulate specific signaling processes, particularly within stem cells.

## Discussion

The early life developmental window represents a crucial time for microbe-host interactions that impacts health both in the short and longer term. Understanding how specific microbiota members modulate host responses in pre-clinical models may help the design and development of next-stage targeted microbiota therapies in humans. Here we investigated how *B*. *breve* UCC2003 induces genome-wide transcriptomic changes in small intestine IECs of neonatal mice. We observed that *B*. *breve* had a global impact on the IEC transcriptome, evidenced by the large number of significantly up-regulated genes and pathways related to cell differentiation and cell proliferation, including genes associated with epithelial barrier function. We propose that *B*. *breve* may act as a key early life microbiota member driving fundamental cellular responses in murine IECs, particularly within the stem cell compartment, and thus drives epithelial barrier development and maintenance during neonatal life stages. However, further clinical studies would be required to determine if our findings extrapolate to the human setting.

*B*. *breve* is known to confer beneficial effect on gut health; however, our knowledge related to the mechanisms underlying these responses is limited. Most studies have focused on targeted immune cells or pathways (during disease and/or inflammation), and to our knowledge no studies have probed global transcriptomic changes within IECs, the frontline physical barrier between bacteria and host (Turroni et al., 2014; Gann, 2010). Our presented findings in a pre-clinical model, namely, ∼4,000 up-regulated DEGs and ∼450 down-regulated DEGs within the *B*. *breve* group, indicate that this *Bifidobacterium* strain modulates whole-scale changes within this critical single-cell layer. Notably, we also examined how *B*. *breve* modulates adult IEC responses; however, we did not observe any significantly differentially regulated genes when compared with control animals. The striking differences in DEGs between these two life points indicate that *B*. *breve* modulation of IECs is limited to the neonatal window. Dominance of *Bifidobacterium* in early life (including strains of *B*. *breve*) overlaps with the development and maturation of many host responses, including epithelial barrier integrity. Therefore, presence of these strains would be expected to play an over-sized role in this initial homeostatic priming, which may afford protection against inflammatory insults in later life, as has been shown previously in a mouse model of pathological epithelial cell shedding (Hughes et al., 2017). Further clinical studies would be required to probe these findings in detail to determine their importance during healthy infant development.

Exploring the murine transcriptional responses in more detail revealed that expressions of key genes associated with formation of epithelial barrier components were up-regulated, including major cell junction protein-encoding genes (75%; 42/56 genes). More specifically, several integrin-associated genes were up-regulated in the presence of UCC2003. Integrins facilitate cell-cell and cell-extracellular matrix adhesion and binding and assembly of the fibronectin matrix that is pivotal for cell migration and cell differentiation (Harburger and Calderwood, 2009; Qin et al., 2004; Mosher et al., 1991). Integrins also play an important role in downstream intracellular signaling that controls cell differentiation, proliferation, and cell survival, including the Raf-MEK-ERK signaling pathway (we also observed enrichment of genes involved in this pathway) (Chernyavsky et al., 2005; Li et al., 2016). Another key intestinal barrier component is represented by TJs, linking complexes between intercellular spaces, and comprise transmembrane proteins including occludins, claudins, zona occludens, and junctional adhesion molecules (Edelblum and Turner, 2009; Groschwitz and Hogan, 2009). Dysfunctional TJ may lead to a “leaky” gut, which is characteristic of numerous intestinal disorders including inflammatory bowel diseases (Krug et al., 2014). Notably, previous work has suggested early life microbiota disruptions (via antibiotic usage) and reductions in *Bifidobacterium* are correlated with increased risk and/or symptoms of ulcerative colitis and Crohn's disease (Kronman et al., 2012; Hildebrand et al., 2008; Favier et al., 1997; Shaw et al., 2010; Ng et al., 2011). Several clinical studies have indicated that supplementation with certain *Bifidobacterium* strains positively modulate gastrointestinal symptoms of patients, which is corrected with reductions of inflammatory markers in colonic IEC-containing biopsies; however, *B*. *breve* UCC2003 has not been used clinically in this patient setting (Furrie et al., 2005; Steed et al., 2010). Similar findings have also been reported in different animal models of intestinal inflammation (Philippe et al., 2011; Grimm et al., 2015; Zuo et al., 2014). A wide range of TJ-related genes were up-regulated after UCC2003 supplementation, particularly *Tjp1* (that encodes ZO-1), *Jam2,* and *Claudin34c1*, with a previous study indicating that other *Bifidobacterium* species (i.e., *B*. *bifidum*) also modulate TJ expression via ZO-1 (Din et al., 2020). These data indicated that specific strains of *Bifidobacterium* may modulate key barrier integrity systems during the neonatal period, and therefore absence of this key initial bacterial-host cross talk may correlate with increased risk of chronic intestinal disorders in later life (Shaw et al., 2010). Intestinal mucus, encoded by *Muc* genes (up-regulated due to *B*. *breve* UCC2003 in this study), plays a crucial role in colonic protection via formation of a physical barrier between the gut lumen and IECs, and deficiencies in MUC-2 have been linked with experimental colitis and increased inflammation in patients with inflammatory bowel disease (Shirazi et al., 2000, Van der Sluis et al., 2006). We have also observed that *B*. *breve* UCC2003 significantly increases goblet cell numbers and mucus production (in gnotobiotic and SPF mice; data not shown). Although the mucus layer may impact direct *Bifidobacterium*-IEC interactions, previous studies have indicated that *B*. *breve* UCC2003 surface molecules, such as EPS and the Tad pilus, may modulate IEC function via signaling through Toll-like receptors (TLRs) (O'Connell Motherway et al., 2019; Hughes et al., 2017). Moreover, bifidobacterial metabolites, such as short-chain fatty acids may also act to modulate the IEC transcriptome, with previous studies indicating enhanced expression of TJs and cadherins via acetate (Hsieh et al., 2015; Ling et al., 2016; Ewaschuk et al., 2008; Lewis et al., 2017).

Further network and functional analysis indicated that clusters of up-regulated DEGs were associated with cell maturation and cell differentiation (as confirmed by cell type-specific analysis), suggesting that neonatal *B*. *breve* exposure positively modulates IEC cell differentiation, growth, and maturation. Somewhat surprisingly, we did not observe the same type of striking responses in immune pathways, which may be masked by the sheer number of DEGs involved in cellular differentiation and processes. However, pathways such as TLR1 and TLR2 pathways do appear to be enriched (cluster 2 of signaling network analysis). This may link to previous work indicating that the UCC2003 EPS signals via TLR2 to induce MyD88 signaling cascades to protect IECs during intestinal inflammation (Hughes et al., 2017). *B*. *breve* M-16V was also shown to interact with TLR2 to up-regulate ubiquitin-editing enzyme A20 expression that correlated with increased tolerance to a TLR4 cascade in porcine IECs, further supporting the involvement of *B*. *breve* in programming key host immunoregulation receptors (Tomosada et al., 2013).

Cell type-specific analysis of DEGs revealed stem cells as the IEC type most affected by *B*. *breve*, with absorptive enterocytes least affected despite being most accessible to bacteria in the gut. It could be hypothesized that *B*. *breve* or their secreted metabolites may reach the crypts of the small intestinal epithelium. This has been previously suggested by *in situ* hybridization histology *in vivo* and by *Bifidobacterium*-conditioned media altering the expression of hundreds of host epithelial genes linked to immune response, cell adhesion, cell cycle, and development in IECs *in vitro* (Hughes et al., 2017; Guo et al., 2015). However, the direct impact of bifidobacterial-associated metabolites on these responses would require further studies to confirm metabolic activity of *B*. *breve* within the small intestine (via transcriptomics and metabolomics), although daily supplementation with live bacteria may also provide a source of these metabolites in our model. Interestingly, certain *Bifidobacterium* and *Lactobacillus* strains that have been heat killed have also been shown to induce host responses, indicating that surface structures alone may play a role in downstream effects (Pique et al., 2019). All but two of the 37 differentially expressed stem cell marker genes were up-regulated in the presence of *B*. *breve* UCC2003, indicating an activating effect resulting in increased pluripotency of stem cells, increased quantity of stem cells, and/or an increased quantity of semi-differentiated cells. Single-cell sequencing of IECs could be used to further investigate this finding. Thirty-two TFs were predicted to regulate these stem cell signature genes, providing possible targets for future investigation of the mechanisms underlying these responses. Functional analysis of the stem cell signature genes and their regulators suggests that *B*. *breve* increases pluripotency of stem cells and/or semi-differentiated epithelial cells through WNT signaling and nuclear hormone signaling (Jeong and Mangelsdorf, 2009). Furthermore, the overrepresentation of the process “RUNX1 regulates transcription of genes involved in differentiation of HSCs” indicates a possible role for histone methylation in response to *B*. *breve* UCC2003 (Imperato et al., 2015). Further determination of host and bacterial metabolome and proteome after *B*. *breve* exposure may allow identification of the specific underlying molecular mechanisms (Guo et al., 2015).

In conclusion, *B*. *breve* UCC2003 plays a central role in orchestrating global neonatal IEC gene responses in a distinct manner as shown in our murine model, modulating genes involved in epithelial barrier development, and driving universal transcriptomic alteration that facilitates cell replication, differentiation, and growth, particularly within the stem cell compartment. This study enhances our overall understanding of the benefits of specific early life microbiota members in intestinal epithelium development, with prospective avenues to probe further health-promoting mechanisms of *Bifidobacterium* in humans. Further work exploring time-dependent transcriptional responses and impact of other *Bifidobacterium* species and strains (and use of mutants and transcriptionally active strains as positive controls), in tandem with metabolomic and proteomic approaches, is required to advance our understanding on the key host pathways and bifidobacterial molecules governing development and maturation of the intestinal barrier during the early life window. Nevertheless, further clinical studies would be essential to explore if these responses and findings are similar to those observed in humans.

### Limitations of the Study

As we only observed low relative abundance of *Bifidobacterium* in our control neonatal animals this may suggest that induction of responses may be linked to the introduction of a new microbiota member (i.e., *B*. *breve* UCC2003), therefore results should be carefully interpreted. However, we did not observe associated global transcriptional inflammatory immune changes that would be expected if this was the case, but rather global changes in barrier function transcripts and pathways. Furthermore, *Bifidobacterium* has previously been isolated from C57BL/6 mice (including from our mouse colony), and therefore appears to be a resident rodent gut microbiota member, although it is found at varying abundances in different animal units and suppliers (Grimm et al., 2015; Hughes et al., 2020). Indeed, one particular study has shown that high levels of resident *Bifidobacterium* in mice directly correlated with improved immune responses to cancer immunotherapies (Sivan et al., 2015). In addition, we did not explore if *B*. *breve* UCC2003 is potentially driving more nuanced microbe-microbe interactions, and that, indirectly, these may also be stimulating IEC responses. Therefore, further studies probing these aspects in more detail, and comparing other *Bifidobacterium* strains, to compare and contrast responses, would be of interest.

*B*. *breve* UCC2003 is a model strain that was previously isolated from the stool of a breast-fed infant (National Collection of Industrial Food and Marine Bacteria, 2020, Sheehan et al., 2007). Although a human-associated strain, it has not been used in clinical studies, so directly extrapolating to human-specific settings should be cautiously considered. Further large-scale clinical studies would be required to confirm any positive strain-level impacts; however, in-depth analysis of, e.g., small IECs would be unethical in a healthy infant cohort, which emphasizes the importance of preclinical models.

Previous studies have shown that this strain can efficiently colonize (long term) the mouse gastrointestinal tract; however, we could not confirm this in our short-term, daily supplementation study (Cronin et al., 2008, O'Connell Motherway et al., 2011). Therefore the IEC responses observed may occur as a result of transient interactions with *B*. *breve* UCC2003 as it passes through the small intestine. Nevertheless, although at lower levels (∼10^5^ CFU/g), we did observe viable *B*. *breve* UCC2003 in the small intestine, linking to our subsequent observations of significant impacts on the IEC transcriptome from this intestinal region.

Very-low-abundance microbiota members (<2% relative abundance), including *Streptococcus*, *Ruminococcus*, *Prevotella*, and *Coprococcus,* were significantly reduced in relative abundance compared with controls, raising the question whether supplementation of *Bifidobacterium* could have reduced these taxa. Regrettably, we could not determine if this is a bifidobacterial effect due to the lack of longitudinal samples, and we did not quantify bacterial titers, which is an important consideration for future work. We also did not profile microbial community composition within the small intestines, which is known to differ from fecal samples.

### Resource Availability

#### Lead Contact

Further information and requests for resources and reagents should be directed to and will be fulfilled by the Lead Contact, Lindsay J. Hall (Lindsay.Hall@quadram.ac.uk).

#### Materials Availability

This study did not generate new unique reagents.

#### Data and Code Availability

The code generated for RNA-seq analysis during this study is available at GitHub https://github.com/raymondkiu/Bifidobacterium-IEC-transcriptomics. The accession number for the raw sequencing reads (both RNA-seq and 16S rRNA amplicon sequencing) reported in this paper is European Nucleotide Archive (ENA): PRJEB36661.

## Methods

All methods can be found in the accompanying Transparent Methods supplemental file.

## Supplemental Information

Document S1. Transparent Methods, Figure S1, Table S3–S9, and S12

Table S1. Highly Expressed Genes (n = 12,965 Genes), Related to Figure 3

Table S2. Significantly Regulated Genes (n = 4,461 Genes), Related to Figure 4

Table S10. Cluster Analysis on DEGs and Related Genes, Related to Figure 5

Table S11. Overlap between Cell Type Signature Genes and Differentially Expressed Genes, Related to Figure 5

## References

[bib1] Arrieta M.C., Stiemsma L.T., Amenyogbe N., Brown E.M., Finlay B. (2014). The intestinal microbiome in early life: health and disease. Front. Immunol..

[bib2] Baucells B.J., Mercadal Hally M., Alvarez Sanchez A.T., Figueras Aloy J. (2016). Probiotic associations in the prevention of necrotising enterocolitis and the reduction of late-onset sepsis and neonatal mortality in preterm infants under 1,500g: a systematic review. An. Pediatr. (Barc.).

[bib3] Chernyavsky A.I., Arredondo J., Karlsson E., Wessler I., Grando S.A. (2005). The Ras/Raf-1/MEK1/ERK signaling pathway coupled to integrin expression mediates cholinergic regulation of keratinocyte directional migration. J. Biol. Chem..

[bib4] Cronin M., Sleator R.D., Hill C., Fitzgerald G.F., van Sinderen D. (2008). Development of a luciferase-based reporter system to monitor Bifidobacterium breve UCC2003 persistence in mice. BMC Microbiol..

[bib5] Derrien M., Alvarez A.S., de Vos W.M. (2019). The gut microbiota in the first decade of life. Trends Microbiol..

[bib6] Din A.U., Hassan A., Zhu Y. (2020). Inhibitory effect of Bifidobacterium Bifidum ATCC29521on colitis and its mechanism. J. Nutr. Biochem..

[bib7] Dominguez-Bello M.G., Costello E.K., Contreras M., Magris M., Hidalgo G., Fierer N., Knight R. (2010). Delivery mode shapes the acquisition and structure of the initial microbiota across multiple body habitats in newborns. Proc. Natl. Acad. Sci. U S A.

[bib8] Edelblum K.L., Turner J.R. (2009). The tight junction in inflammatory disease: communication breakdown. Curr. Opin. Pharmacol..

[bib9] Ewaschuk J.B., Diaz H., Meddings L., Diederichs B., Dmytrash A., Backer J., Looijer-van Langen M., Madsen K.L. (2008). Secreted bioactive factors from Bifidobacterium infantis enhance epithelial Cell barrier function. Am. J. Physiol. Gastrointest. Liver Physiol..

[bib10] Favier C., Neut C., Mizon C., Cortot A., Colombel J.F., Mizon J. (1997). Fecal beta-D-galactosidase production and Bifidobacteria are decreased in Crohn's disease. Dig. Dis. Sci..

[bib11] Fukuda S., Toh H., Taylor T.D., Ohno H., Hattori M. (2012). Acetate-producing bifidobacteria protect the host from enteropathogenic infection via carbohydrate transporters. Gut Microbes.

[bib12] Furrie E., Macfarlane S., Kennedy A., Cummings J.H., Walsh S.V., O'neil D.A., Macfarlane G.T. (2005). Synbiotic therapy (Bifidobacterium longum/Synergy 1) initiates resolution of inflammation in patients with active ulcerative colitis: a randomised controlled pilot trial. Gut.

[bib13] Gann R.N. (2010). Host Signaling Response to Adhesion of Bifidobacterium infantis.

[bib14] Garcia-Alonso L., Holland C.H., Ibrahim M.M., Turei D., Saez-Rodriguez J. (2019). Benchmark and integration of resources for the estimation of human transcription factor activities. Genome Res..

[bib15] Grimm V., Radulovic K., Riedel C.U. (2015). Colonization of C57BL/6 mice by a potential probiotic Bifidobacterium bifidum strain under germ-free and specific pathogen-free conditions and during experimental colitis. PLoS One.

[bib16] Groschwitz K.R., Hogan S.P. (2009). Intestinal barrier function: molecular regulation and disease pathogenesis. J. Allergy Clin. Immunol..

[bib17] Guo S., Guo Y., Ergun A., Lu L., Walker W.A., Ganguli K. (2015). Secreted metabolites of Bifidobacterium infantis and Lactobacillus acidophilus protect immature human enterocytes from IL-1beta-induced inflammation: a transcription profiling analysis. PLoS One.

[bib18] Haber A.L., Biton M., Rogel N., Herbst R.H., Shekhar K., Smillie C., Burgin G., Delorey T.M., Howitt M.R., Katz Y., Tirosh I., Beyaz S., Dionne D., Zhang M. (2017). A single-cell survey of the small intestinal epithelium. Nature.

[bib19] Harburger D.S., Calderwood D.A. (2009). Integrin signalling at a glance. J. Cell Sci..

[bib20] Hildebrand H., Malmborg P., Askling J., Ekbom A., Montgomery S.M. (2008). Early-life exposures associated with antibiotic use and risk of subsequent Crohn's disease. Scand. J. Gastroenterol..

[bib21] Holland C.H., Szalai B., Saez-Rodriguez J. (2019). Transfer of regulatory knowledge from human to mouse for functional genomics analysis. Biochim. Biophys. Acta Gene Regul. Mech..

[bib22] Hsieh C.Y., Osaka T., Moriyama E., Date Y., Kikuchi J., Tsuneda S. (2015). Strengthening of the intestinal epithelial tight junction by Bifidobacterium bifidum. Physiol. Rep..

[bib23] Hughes K.R., Harnisch L.C., Alcon-Giner C., Mitra S., Wright C.J., Ketskemety J., van Sinderen D., Watson A.J., Hall L.J. (2017). Bifidobacterium breve reduces apoptotic epithelial cell shedding in an exopolysaccharide and MyD88-dependent manner. Open Biol..

[bib24] Hughes K.R., Schofield Z., Dalby M.J., Caim S., Chalklen L., Bernuzzi F., Alcon-Giner C., Le Gall G., Watson A.J.M., Hall L.J. (2020). The early life microbiota protects neonatal mice from pathological small intestinal epithelial cell shedding. FASEB J..

[bib25] Imperato M.R., Cauchy P., Obier N., Bonifer C. (2015). The RUNX1-PU.1 axis in the control of hematopoiesis. Int. J. Hematol..

[bib26] Jacobs S.E., Tobin J.M., Opie G.F., Donath S., Tabrizi S.N., Pirotta M., Morley C.J., Garland S.M., Proprems Study G. (2013). Probiotic effects on late-onset sepsis in very preterm infants: a randomized controlled trial. Pediatrics.

[bib27] Jeong Y., Mangelsdorf D.J. (2009). Nuclear receptor regulation of stemness and stem cell differentiation. Exp. Mol. Med..

[bib28] Kronman M.P., Zaoutis T.E., Haynes K., Feng R., Coffin S.E. (2012). Antibiotic exposure and IBD development among children: a population-based cohort study. Pediatrics.

[bib29] Krug S.M., Schulzke J.D., Fromm M. (2014). Tight junction, selective permeability, and related diseases. Semin. Cell Dev. Biol..

[bib30] Lawson M.A.E., O'neill I.J., Kujawska M., Gowrinadh Javvadi S., Wijeyesekera A., Flegg Z., Chalklen L., Hall L.J. (2020). Breast milk-derived human milk oligosaccharides promote Bifidobacterium interactions within a single ecosystem. ISME J..

[bib31] Lewis M.C., Merrifield C.A., Berger B., Cloarec O., Duncker S., Mercenier A., Nicholson J.K., Holmes E., Bailey M. (2017). Early intervention with Bifidobacterium lactis NCC2818 modulates the host-microbe interface independent of the sustained changes induced by the neonatal environment. Sci. Rep..

[bib32] Li L., Zhao G.D., Shi Z., Qi L.L., Zhou L.Y., Fu Z.X. (2016). The Ras/Raf/MEK/ERK signaling pathway and its role in the occurrence and development of HCC. Oncol. Lett..

[bib33] Ling X., Linglong P., Weixia D., Hong W. (2016). Protective effects of Bifidobacterium on intestinal barrier function in LPS-induced enterocyte barrier injury of caco-2 monolayers and in a rat NEC model. PLoS One.

[bib34] Liu C., Zhang Z.Y., Dong K., Guo X.K. (2010). Adhesion and immunomodulatory effects of Bifidobacterium lactis HN019 on intestinal epithelial cells INT-407. World J. Gastroenterol..

[bib35] Mikami K., Kimura M., Takahashi H. (2012). Influence of maternal bifidobacteria on the development of gut bifidobacteria in infants. Pharmaceuticals (Basel).

[bib36] Mosher D.F., Fogerty F.J., Chernousov M.A., Barry E.L. (1991). Assembly of fibronectin into extracellular matrix. Ann. N. Y Acad. Sci..

[bib37] Nagpal R., Kurakawa T., Tsuji H., Takahashi T., Kawashima K., Nagata S., Nomoto K., Yamashiro Y. (2017). Evolution of gut Bifidobacterium population in healthy Japanese infants over the first three years of life: a quantitative assessment. Sci. Rep..

[bib38] National Collection of Industrial Food and Marine Bacteria (NCIMB) (2020). NCIMB 8807 General Info.

[bib39] Ng S.C., Benjamin J.L., Mccarthy N.E., Hedin C.R., Koutsoumpas A., Plamondon S., Price C.L., Hart A.L., Kamm M.A., Forbes A., Knight S.C., Lindsay J.O., Whelan K., Stagg A.J. (2011). Relationship between human intestinal dendritic cells, gut microbiota, and disease activity in Crohn's disease. Inflamm. Bowel Dis..

[bib40] Nusse R. (2008). Wnt signaling and stem cell control. Cell Res..

[bib41] O'Connell Motherway M., Houston A., O'callaghan G., Reunanen J., O'brien F., O'driscoll T., Casey P.G., de Vos W.M., van Sinderen D., Shanahan F. (2019). A Bifidobacterial pilus-associated protein promotes colonic epithelial proliferation. Mol. Microbiol..

[bib42] O'Connell Motherway M., Zomer A., Leahy S.C., Reunanen J., Bottacini F., Claesson M.J., O'brien F., Flynn K., Casey P.G., Munoz J.A., Kearney B., Houston A.M., O'mahony C. (2011). Functional genome analysis of Bifidobacterium breve UCC2003 reveals type IVb tight adherence (Tad) pili as an essential and conserved host-colonization factor. Proc. Natl. Acad. Sci. U S A.

[bib43] O'Neill I., Schofield Z., Hall L.J. (2017). Exploring the role of the microbiota member Bifidobacterium in modulating immune-linked diseases. Emerg. Top. Life Sci..

[bib44] Pan W.H., Sommer F., Falk-Paulsen M., Ulas T., Best P., Fazio A., Kachroo P., Luzius A., Jentzsch M., Rehman A., Muller F., Lengauer T., Walter J., Kunzel S., Baines J.F. (2018). Exposure to the gut microbiota drives distinct methylome and transcriptome changes in intestinal epithelial cells during postnatal development. Genome Med..

[bib45] Patole S.K., Rao S.C., Keil A.D., Nathan E.A., Doherty D.A., Simmer K.N. (2016). Benefits of Bifidobacterium breve M-16V supplementation in preterm neonates - a retrospective cohort study. PLoS One.

[bib46] Philippe D., Heupel E., Blum-Sperisen S., Riedel C.U. (2011). Treatment with Bifidobacterium bifidum 17 partially protects mice from Th1-driven inflammation in a chemically induced model of colitis. Int. J. Food Microbiol..

[bib47] Pique N., Berlanga M., Minana-Galbis D. (2019). Health benefits of heat-killed (Tyndallized) probiotics: an overview. Int. J. Mol. Sci..

[bib48] Plantinga T.S., van Maren W.W., van Bergenhenegouwen J., Hameetman M., Nierkens S., Jacobs C., de Jong D.J., Joosten L.A., Van't Land B., Garssen J., Adema G.J., Netea M.G. (2011). Differential Toll-like receptor recognition and induction of cytokine profile by Bifidobacterium breve and Lactobacillus strains of probiotics. Clin. Vaccin. Immunol..

[bib49] Plaza-Diaz J., Gomez-Llorente C., Fontana L., Gil A. (2014). Modulation of immunity and inflammatory gene expression in the gut, in inflammatory diseases of the gut and in the liver by probiotics. World J. Gastroenterol..

[bib50] Plummer E.L., Bulach D.M., Murray G.L., Jacobs S.E., Tabrizi S.N., Garland S.M., Proprems Study G. (2018). Gut microbiota of preterm infants supplemented with probiotics: sub-study of the ProPrems trial. BMC Microbiol..

[bib51] Qin J., Vinogradova O., Plow E.F. (2004). Integrin bidirectional signaling: a molecular view. PLoS Biol..

[bib52] Riedel C.U., Foata F., Philippe D., Adolfsson O., Eikmanns B.J., Blum S. (2006). Anti-inflammatory effects of bifidobacteria by inhibition of LPS-induced NF-kappaB activation. World J. Gastroenterol..

[bib53] Robertson C., Savva G.M., Clapuci R., Jones J., Maimouni H., Brown E., Minocha A., Hall L.J., Clarke P. (2020). Incidence of necrotising enterocolitis before and after introducing routine prophylactic Lactobacillus and Bifidobacterium probiotics. Arch. Dis. Child. Fetal Neonatal Ed..

[bib54] Shaw S.Y., Blanchard J.F., Bernstein C.N. (2010). Association between the use of antibiotics in the first year of life and pediatric inflammatory bowel disease. Am. J. Gastroenterol..

[bib55] Sheehan V.M., Sleator R.D., Hill C., Fitzgerald G.F. (2007). Improving gastric transit, gastrointestinal persistence and therapeutic efficacy of the probiotic strain Bifidobacterium breve UCC2003. Microbiology.

[bib56] Shirazi T., Longman R.J., Corfield A.P., Probert C.S. (2000). Mucins and inflammatory bowel disease. Postgrad. Med. J..

[bib57] Sivan A., Corrales L., Hubert N., Williams J.B., Aquino-Michaels K., Earley Z.M., Benyamin F.W., Lei Y.M., Jabri B., Alegre M.L., Chang E.B., Gajewski T.F. (2015). Commensal Bifidobacterium promotes antitumor immunity and facilitates anti-PD-L1 efficacy. Science.

[bib58] Srutkova D., Schwarzer M., Hudcovic T., Zakostelska Z., Drab V., Spanova A., Rittich B., Kozakova H., Schabussova I. (2015). Bifidobacterium longum CCM 7952 promotes epithelial barrier function and prevents acute DSS-induced colitis in strictly strain-specific manner. PLoS One.

[bib59] Steed H., Macfarlane G.T., Blackett K.L., Bahrami B., Reynolds N., Walsh S.V., Cummings J.H., Macfarlane S. (2010). Clinical trial: the microbiological and immunological effects of synbiotic consumption - a randomized double-blind placebo-controlled study in active Crohn's disease. Aliment. Pharmacol. Ther..

[bib60] Stewart C.J., Ajami N.J., O'brien J.L., Hutchinson D.S., Smith D.P., Wong M.C., Ross M.C., Lloyd R.E., Doddapaneni H., Metcalf G.A., Muzny D., Gibbs R.A., Vatanen T., Huttenhower C., Xavier R.J., Rewers M. (2018). Temporal development of the gut microbiome in early childhood from the TEDDY study. Nature.

[bib61] Thoo L., Noti M., Krebs P. (2019). Keep calm: the intestinal barrier at the interface of peace and war. Cell Death Dis..

[bib62] Tomosada Y., Villena J., Murata K., Chiba E., Shimazu T., Aso H., Iwabuchi N., Xiao J.Z., Saito T., Kitazawa H. (2013). Immunoregulatory effect of bifidobacteria strains in porcine intestinal epithelial cells through modulation of ubiquitin-editing enzyme A20 expression. PLoS One.

[bib63] Turroni F., Taverniti V., Ruas-Madiedo P., Duranti S., Guglielmetti S., Lugli G.A., Gioiosa L., Palanza P., Margolles A., van Sinderen D., Ventura M. (2014). Bifidobacterium bifidum PRL2010 modulates the host innate immune response. Appl. Environ. Microbiol..

[bib64] Van der Sluis M., de Koning B.A., de Bruijn A.C., Velcich A., Meijerink J.P., van Goudoever J.B., Buller H.A., Dekker J., van Seuningen I., Renes I.B., Einerhand A.W. (2006). Muc2-deficient mice spontaneously develop colitis, indicating that MUC2 is critical for colonic protection. Gastroenterology.

[bib65] Zheng B., van Bergenhenegouwen J., Overbeek S., van de Kant H.J., Garssen J., Folkerts G., Vos P., Morgan M.E., Kraneveld A.D. (2014). Bifidobacterium breve attenuates murine dextran sodium sulfate-induced colitis and increases regulatory T cell responses. PLoS One.

[bib66] Zuo L., Yuan K.T., Yu L., Meng Q.H., Chung P.C., Yang D.H. (2014). Bifidobacterium infantis attenuates colitis by regulating T cell subset responses. World J. Gastroenterol..

