# Supplementary material for: Bifidobacterium breve UCC2003 Induces a Distinct Global Transcriptomic Program in Neonatal Murine Intestinal Epithelial Cells
Source: iScience. 2020 Jul 2;23(7):101336. doi: 10.1016/j.isci.2020.101336 (PMC7371750; doi:10.1016/j.isci.2020.101336)
Supplement: Document S1. Transparent Methods, Figure S1, Table S3–S9, and S12 [file mmc1.pdf]

iScience, Volume *23*

Supplemental Information

Bifidobacterium breve UCC2003 Induces a Distinct Global Transcriptomic Program in Neonatal Murine Intestinal Epithelial Cells

Raymond Kiu, Agatha Treveil, Lukas C. Harnisch, Shabhonam Caim, Charlotte Leclaire, Douwe van Sinderen, Tamas Korcsmaros, and Lindsay J. Hall

SUPPLEMENTAL FIGURE

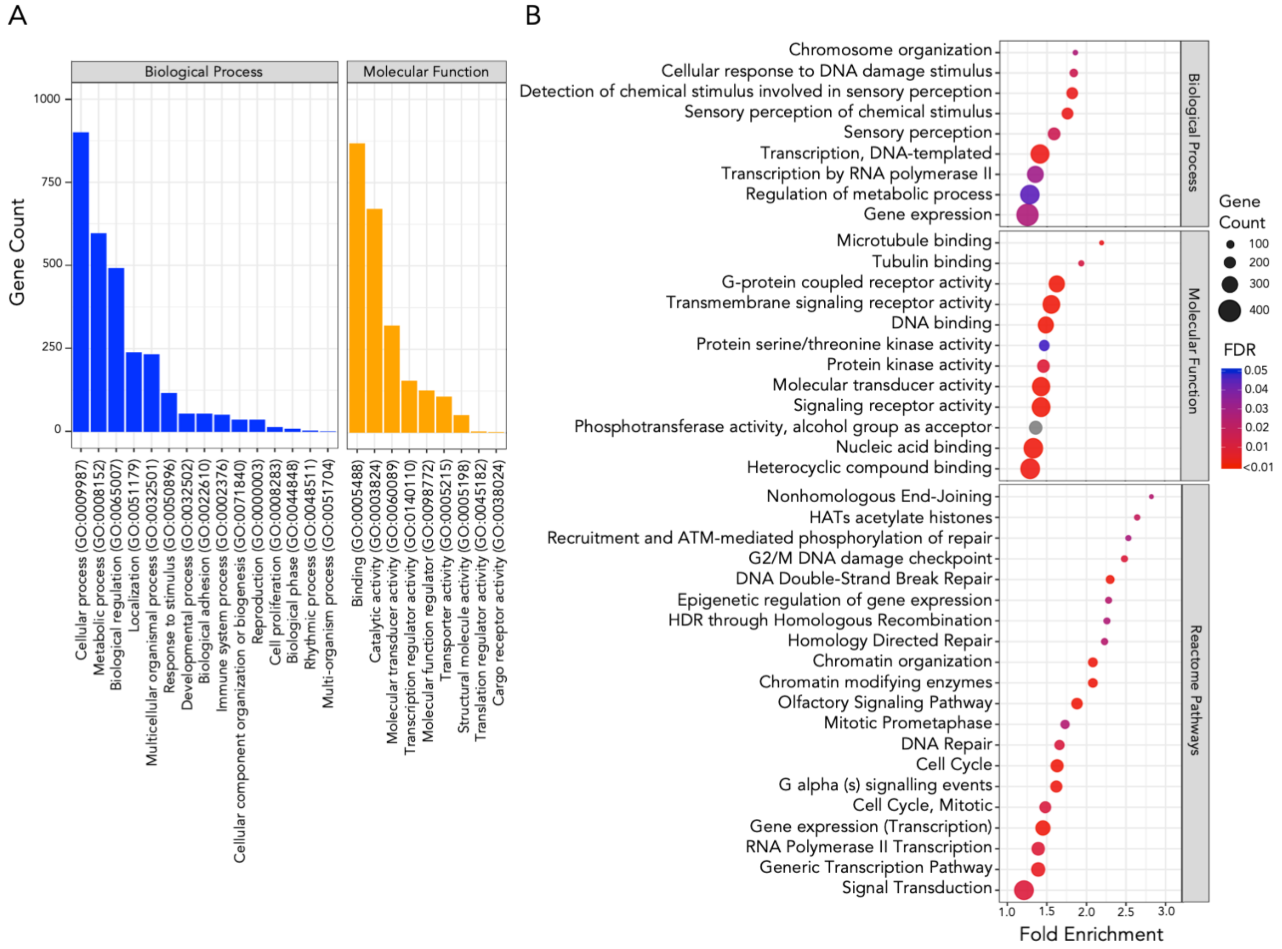

Figure S1. Functional analysis on differentially expressed genes. Related to Figure 4.
(A) Panther Slim GO-term major categories of significantly up-regulated genes (*n*=3,996). Related to Table S7 and Table S8.
(B) Functional and pathway enrichment analysis on significantly up-regulated genes (Panther Slim GO-term). Only top 20 FDR-ranked enriched pathways (Reactome pathways) are shown. Statistical significance cut-offs: FDR<0.05. Statistical significance: Fisher's Exact Test. Fold Enrichment was calculated against all expressed genes in IECs as the background (*n*=21,537). Related to Table S5, Table S6 and Table S9.

SUPPLEMENTAL TABLES

Gene	Ensembl ID	Chromosome	Description/ Putative function	Biological process (GO)
Gm27149	ENSMUSG00000098426	7	Unknown	Unknown
Ccnb1ip1	ENSMUSG00000071470	14	Cyclin B1 interacting protein 1	Cell differentiation, Cellular component organisation, Protein metabolic process
Gm10359	ENSMUSG00000094708	5	glyceraldehyde-3-phosphate dehydrogenase pseudogene	Unknown
Gm12671	ENSMUSG00000095937	4	glyceraldehyde-3-phosphate dehydrogenase pseudogene	Unknown
Gm48216	ENSMUSG00000114367	13	Unknown	Unknown
Gm17131	ENSMUSG00000085328	5	Unknown	Unknown
Tmem72	ENSMUSG00000048108	6	transmembrane protein 72	Unknown
Ccdc107	ENSMUSG00000028461	4	coiled-coil domain containing 107	Unknown
CR936839.3	ENSMUSG00000111855	Unknown	Unknown	Unknown
Hist1h4b	ENSMUSG00000069266	13	H4 clustered histone 2	Cell differentiation, Cellular component organisation, Immune system process, system development
Gm48836	ENSMUSG00000113523	12	Unknown	Unknown
Gm42669	ENSMUSG00000106631	5	Unknown	Unknown
Gprin3	ENSMUSG00000045441	6	GPRIN family member 3	Unknown
Fgd4	ENSMUSG00000022788	16	FYVE, RhoGEF and PH domain containing 4	Cellular component of organisation, Protein metabolic process, Response to stimulus, Signaling
D10Wsu102e	ENSMUSG00000020255	10	DNA segment, Chr 10, Wayne State University 102, expressed	Unknown
Gm20594	ENSMUSG00000096887	6	Unknown	Cell death, Response to stimulus, Signaling
Vps13b	ENSMUSG00000037646	15	vacuolar protein sorting 13B	Establishment of localisation
Gm48366	ENSMUSG00000113523	12	Unknown	Unknown
Gm48054	ENSMUSG00000113921	13	Unknown	Unknown
Naip6	ENSMUSG00000078942	13	NLR family, apoptosis inhibitory protein 6	Cell death, Immune system process, Response to stimulus

Table S3. Annotation of top 20 significantly up-regulated genes. Related to Figure 4.

Gene	Ensembl ID	Chromosome	Description/ Putative function	Biological process (GO)
Gm7849	ENSMUSG00000079114	8	Defensin	Immune system process, response to stimulus
Rps27rt	ENSMUSG00000050621	9	ribosomal protein S27, retrogene	Unknown
Gm6158	ENSMUSG00000090381	14	Unknown	Unknown
mt-Co2	ENSMUSG00000064354	MT	mitochondrially encoded cytochrome c oxidase II	Carbohydrate derivative metabolism, cell death
Gm3650	ENSMUSG00000097891	18	Unknown	Unknown
Gm5831	ENSMUSG00000111133	9	Unknown	Unknown
Gm7331	ENSMUSG00000059461	X	Unknown	Unknown
Polr3h	ENSMUSG00000022476	15	polymerase (RNA) III (DNA directed) polypeptide H	Immune system process, response to stimulus
Gm14698	ENSMUSG00000071748	X	Unknown	Unknown
Ftl1-ps1	ENSMUSG00000062382	13	erritin light polypeptide 1	Unknown
Rps16	ENSMUSG00000037563	7	ribosomal protein S16	Protein metabolic process, response to stimulus
Mt2	ENSMUSG00000031762	8	metallothionein 2	Homeostatic process, response to stimulus, signaling
Mt1	ENSMUSG00000031765	8	metallothionein 1	Cell death, homeostatic process, response to stimulus, signaling
Hba-a1	ENSMUSG00000069919	11	hemoglobin alpha	Cell differentiation, homeostatic process, immune system process, response to stimulus, signaling, system development
Hbb-bt	ENSMUSG00000073940	7	hemoglobin, beta adult t chain	Unknown
Rps2-ps10	ENSMUSG00000091957	18	ribosomal protein S2	Unknown
Gpx3	ENSMUSG00000018339	11	glutathione peroxidase 3	Response to stimulus
Smcp	ENSMUSG00000074435	3	sperm mitochondria-associated cysteine-rich protei	Unknown
Gm49384	ENSMUSG00000113786	12	Predicted gene	Unknown
Gm7336	ENSMUSG00000078636	7	Predicted gene	Unknown

Table S4. Annotation of top 20 significantly down-regulated genes. Related to Figure 4.

GO Biological Process	Gene Count	Fold Enrichment	FDR
Transcription, DNA-templated	252	1.41	1.15E-03
Detection of chemical stimulus involved in sensory perception	97	1.82	1.92E-03
Sensory perception of chemical stimulus	98	1.76	1.97E-03
Cellular response to DNA damage stimulus	66	1.84	1.54E-02
Sensory perception	111	1.59	1.57E-02
Gene expression	350	1.25	2.66E-02
Chromosome organization	57	1.86	2.66E-02
Transcription by RNA polymerase II	193	1.35	3.09E-02
Regulation of metabolic process	262	1.28	4.48E-02

Table S5. GO Biological Process enrichment analysis in up-regulated DEGs. Related to Figure 4 and Figure S1.

GO Molecular Functions	Gene Count	Fold Enrichment	FDR
Transmembrane signaling receptor activity	279	1.56	5.07E-08
G-protein coupled receptor activity	224	1.63	8.61E-08
Molecular transducer activity	321	1.43	1.76E-06
Signaling receptor activity	302	1.43	4.06E-06
DNA binding	225	1.49	1.20E-05
Nucleic acid binding	344	1.33	1.14E-04
Heterocyclic compound binding	352	1.29	7.08E-04
Microtubule binding	43	2.2	3.01E-03
Protein kinase activity	133	1.46	7.97E-03
Tubulin binding	45	1.94	1.24E-02
Protein serine/threonine kinase activity	94	1.47	4.74E-02
Phosphotransferase activity, alcohol group as acceptor	144	1.36	5.04E-02

Table S6. GO Molecular Functions enrichment analysis in up-regulated DEGs. Related to Figure 4 and Figure S1.

Biological Process	Genes	Percentage
cellular process (GO:0009987)	901	31.70%
metabolic process (GO:0008152)	597	21.00%
biological regulation (GO:0065007)	492	17.30%
localization (GO:0051179)	239	8.40%
multicellular organismal process (GO:0032501)	233	8.20%
response to stimulus (GO:0050896)	117	4.10%
developmental process (GO:0032502)	55	1.90%
biological adhesion (GO:0022610)	55	1.90%
immune system process (GO:0002376)	52	1.80%
cellular component organization or biogenesis (GO:0071840)	37	1.30%
reproduction (GO:0000003)	37	1.30%
cell proliferation (GO:0008283)	15	0.50%
biological phase (GO:0044848)	10	0.40%
rhythmic process (GO:0048511)	4	0.10%
multi-organism process (GO:0051704)	2	0.10%

Table S7. GO Biological Process functional assignment to 3,996 upregulated genes. Related to Figure 4 and Figure S1.

Molecular Function	Genes	Percentage
binding (GO:0005488)	868	37.60%
catalytic activity (GO:0003824)	671	29.00%
molecular transducer activity (GO:0060089)	321	13.90%
transcription regulator activity (GO:0140110)	156	6.80%
molecular function regulator (GO:0098772)	127	5.50%
transporter activity (GO:0005215)	108	4.70%
structural molecule activity (GO:0005198)	53	2.30%
translation regulator activity (GO:0045182)	4	0.20%
cargo receptor activity (GO:0038024)	2	0.10%

Table S8. GO Molecular Function functional assignment to 3,996 upregulated genes. Related to Figure 4 and Figure S1.

Reactome Pathway	Gene Count	Fold Enrichment	FDR
Olfactory Signaling Pathway	108	1.88	3.20E-05
Cell Cycle	154	1.63	1.13E-04
Gene expression (Transcription)	238	1.45	1.18E-04
Chromatin organization	67	2.08	2.54E-04
Chromatin modifying enzymeS	67	2.08	2.82E-04
DNA Double-Strand Break Repair	51	2.3	4.19E-04
G alpha (s) signalling events	122	1.62	6.50E-04
RNA Polymerase II Transcription	202	1.39	3.06E-03
Generic Transcription Pathway	173	1.39	8.50E-03
Signal Transduction	461	1.21	8.98E-03
G2/M DNA damage checkpoint	29	2.48	9.07E-03
DNA Repair	77	1.66	1.06E-02
Cell Cycle, Mitotic	122	1.48	1.06E-02
HATs acetylate histones	23	2.64	1.85E-02
Epigenetic regulation of gene expression	28	2.28	2.38E-02
Homology Directed Repair	30	2.23	2.39E-02
Nonhomologous End-Joining	19	2.82	2.46E-02
Mitotic Prometaphase	58	1.73	2.48E-02
HDR through Homologous Recombination	29	2.26	2.63E-02
Recruitment and ATM-mediated phosphorylation of repair	22	2.53	2.78E-02

Table S9. Reactome pathway enrichment analysis in up-regulated DEGs. Related to Figure 4 and Figure S1.

Reactome Pathway	Gene Count	Fold Enrichment	FDR
Regulation of FZD by ubiquitination (R-MMU-4641263)	3	35.6	2.39E-02
Endogenous sterols (R-MMU-211976)	3	27.06	3.63E-02
Estrogen-dependent gene expression (R-MMU-9018519)	6	19.61	4.95E-04
ESR-mediated signaling (R-MMU-8939211)	6	18.04	4.69E-04
RUNX1 regulates transcription of genes involved in differentiation of HSCs (R-MMU-8939236)	4	14.78	3.41E-02
Signaling by Nuclear Receptors (R-MMU-9006931)	6	11.66	4.23E-03
Transcriptional regulation by RUNX1 (R-MMU-8878171)	6	9.73	9.64E-03
Generic Transcription Pathway (R-MMU-212436)	15	5.88	4.71E-05
RNA Polymerase II Transcription (R-MMU-73857)	15	5	1.89E-04
Gene expression (Transcription) (R-MMU-74160)	15	4.38	4.93E-04
Signal Transduction (R-MMU-162582)	19	2.37	3.65E-02

Table S12. Reactome pathway enrichment analysis of differentially expressed stem cell signature genes and their expressed regulators. Related to Figure 5.

TRANSPARENT METHODS

Animals

All animal experiments and related protocols were performed in accordance with the Animals (Scientific Procedures) Act 1986 (ASPA) under project licence (PPL: 80/2545) and personal licence (PIL: I68D4DCCF), approved by UK Home Office and University of East Anglia (UEA) FMH Research Ethics Committee. C57BL/6J two-week-old neonatal female mice (*n*=10) were housed in two separate cages with their mothers within UEA Disease Modelling Unit. Mice were euthanised via ASPA Schedule 1 protocol (CO_2 and cervical dislocation).

Bacterial culturing, inoculum preparation and CFU enumeration

B. breve UCC2003 (also known as NCIMB 8807) was streaked from frozen glycerol stocks onto autoclaved Reinforced Clostridial Agar (RCA) plates (Oxoid, UK) and incubated in an anaerobic chamber (miniMACS, Don Whitley Scientific) at 37°C for 48 h prior to picking single colonies for inoculation in prewarmed sterilised Reinforced Clostridial Medium (Oxoid, UK).

For preparation of gavage inoculums, 5 ml of inoculated broth was incubated overnight followed by sub-culturing into 5 ml De Man, Rogosa and Sharpe (MRS) medium (Oxoid). After an additional overnight incubation, another sub-culturing into 40 ml RCM was performed. Inoculums were prepared from cultures by 3 rounds of centrifugation at 3220 g for 10 min followed by three PBS washes before dilution in 4 ml (adult mice) or 2 ml (neonatal mice) sterile PBS. Bacterial concentration of inoculum was enumerated by plating serial dilutions in sterile PBS on RCA plates and enumerating colonies following two-day incubation to calculate CFU/ml.

Bacterial treatment and administration

Neonatal mice were orally gavaged with *B. breve* UCC2003 inoculations of 10^8 CFU/ml in 50 µl every 24 h for 3 consecutive days. Control mice received oral gavages of sterile PBS. *B. breve* UCC2003 viable presence/transition through the gut was confirmed by collection of fresh faeces or intestinal content homogenised with 1 ml sterile PBS followed by serial-dilution plating in sterile PBS on RCA supplemented with 50 mg/L mupirocin and counting of colonies following two-day incubation to calculate CFU/mg.

Gut microbiota profiling by 16S rRNA amplicon sequencing and analysis

Genomic DNA extraction of mouse faecal samples on day 4 was performed with FastDNA Spin Kit for Soil (MP Biomedicals) following manufacturer's instructions and extending the bead-beating step to 3 min as described previously (Alcon-Giner et al., 2019). Extracted DNA was quantified, normalised and sequenced on Illumina MiSeq platform using a read length of 2 × 300 bp. After quality pre-filtering and removals of chimeras, sequencing reads were analysed using open-reference OTU clustering strategy (QIIME v1.9.1) to assign bacterial taxonomy based on SILVA_132 database (Quast et al., 2013). OTU tables in BIOM format was converted to genus counts in MEGAN6 and visualised using R library *ggplot2* as described previously (Kiu et al., 2019, Caporaso et al., 2010, Huson et al., 2016).

Tissue collection and isolation of small intestinal epithelial cells (IECs)

Upon tissue harvesting, 0.5 cm^2 sections of small intestines were collected in 200 µl RNAlater™ (Thermo Fisher Scientific) at the animal unit prior to IECs isolation (from fresh samples) via an adapted Weisser method as described below (Hughes et al., 2017). Sections of small intestines were placed in ice-cold PBS in 200 ml Duran bottles. Faecal matter was washed off by inverting 10 times in 0.154M NaCl and 1mM DTT. Liquid was drained and mucus layer removed through incubation of samples in 1.5mM KCl, 96mM NaCl, 27 mM Tri-sodium citrate, 8mM NaH_2PO4 and 5.6mM Na2HPO4 for 15 min at 220 rpm and 37°C. IECs were separated from basal membrane by incubation in 1.5 mM EDTA and 0.5 mM DTT for 15 min at 200 rpm and 37 °C followed by shaking vigorously 20 times. IECs were collected from solution by centrifugation at 500 g for 10 min at 4 ˚C. Supernatant was then discarded and cell pellet resuspended in 3 ml of ice-cold PBS. Cell concentrations of

isolated IEC samples calculated by labelling dead cell with trypan blue at a 1:1 v/v ratio and enumeration of viable cells using a Neubauer haemocytometer on an inverted microscope (ID03, Zeiss).

RNA extraction and sequencing

RNA was extracted from IECs by adding a volume containing 2 x 10^6 cells in PBS to QIAshredder spin columns (QIAGEN) followed by centrifugation at 9,300 g for 1 min. Follow-through was mixed with 600 µl RLT lysis buffer and used for subsequent RNA isolation. Homogenised samples in RLT buffer from IECs were processed by adding 700 µl of 70% ethanol and mixing by pipetting. Samples were then added into RNeasy spin column and spun at 8,000 g for 15 s. Flow-through was discarded and process repeated until all of sample was filtered through column. Then 700 µl of buffer RW1 was added to column and centrifuge at 8,000 g for 30 s. Again, flow through was discarded and filter placed in a new collection tube. To the filter, 500 µl RPE was added and spun at 8,000 g for 30 s followed by discarding of flow through. An additional 500 µl RPE was pipetted into column and centrifuged at 8,000 g for 2 min. Spin column was then placed in a new collection tube and centrifuged at 8,000 g for 2 min. Columns were transferred to a RNA low-bind Eppendorf tube and 30 µl of RNase free water added to directly to the filter. After an incubation of 1 min at RT, sample was centrifuged at 8,000 g for 1 min and flow through containing RNA stored at -80˚C.

Purified RNA was quantified, and quality controlled using RNA 6000 Nano kit on a 2100 Bioanalyser (Agilent). Only samples with RIN values above 8 were sequenced. RNA sequencing was performed at the Wellcome Trust Sanger Institute (Hinxton, UK) on paired-end 75 bp inserts on an Illumina HiSeq 2000 platform. Isolated RNA was processed by poly-A selection and/or Ribo-depletion.

Sequence pre-processing and Differential Gene Expression (DGE) analysis

Sequencing quality of raw FASTQ reads were assessed by FastQC software (v0.11.8). FASTQ reads were subsequently quality-filtered using fastp v0.20.0 with options -q 10 (phred quality <10 was discarded) followed by merging reads into single read file for each sample (merge-paired-reads.sh) and rRNA sequence filtering via SortMeRNA v2.1 based on SILVA rRNA database optimised for SortMeRNA software (Chen et al., 2018, Kopylova et al., 2012). Filtered reads were then unmerged (unmerge-paired-reads.sh) and ready for transcript quantification.

Transcript mapping and quantification were performed using Kallisto v0.44.0 (Bray et al., 2016). Briefly, *Mus musculus* (C57BL/6 mouse) cDNA sequences (GRCm38.release-98_k31) were retrieved from Ensembl database and built into an index database with Kallisto utility index at default parameter that was used for following transcript mapping and abundance quantification via Kallisto utility quant at 100 bootstrap replicates (-b 100) (Zerbino et al., 2018).

DGE analysis was performed using R library Sleuth (v0.30.0) (Pimentel et al., 2017). Gene transcripts were mapped to individual genes using Ensembl BioMart database with Sleuth function *sleuth_prep* with option *gene_mode = TRUE*. Genes with an absolute $\log_2$(fold change) >1.0 (based on Wald test statistics) and q value <0.05 (or, FDR-adjusted p value; based on likelihood ratio test) were considered to be significantly regulated (Kinsella et al., 2011).

Functional annotation and enrichment analysis

Functional assignment and enrichment analysis was performed using PANTHER Classification System (Mi et al., 2019a). Briefly, for functional assignment analysis, a list of genes of interest in Ensembl IDs were supplied to the webserver to be mapped to the Mouse Genome Database (MGD) to generate functional classification on those genes of interest (Bult et al., 2019). For functional enrichment analysis, a gene list was supplied together with a background gene list in Ensembl IDs to Panther web server, then selected 'functional overrepresentation test' and chose a particular function class in the drop-down menu. Recommended by the database developers, Fisher's exact test and False Discovery Rate (FDR) correction were used to perform enrichment analysis (Mi et

al., 2019b). FDR <0.05 was used as the default cut-off for significant enrichment. Functional annotation of top 20 up/down-regulated genes was assigned manually via Ensembl and/or MGI (Mouse Genome Informatics) databases (Bult et al., 2019, Cunningham et al., 2019).

Network, cluster and signalling pathway analysis

A signalling network of all up-regulated DEGs and their first neighbours was built using all available biological signalling databases in the Cytoscape (v3.7.2) OmniPath app (v1, *Mus musculus*) (Turei et al., 2016, Shannon et al., 2003). Modules of highly connected genes within the signalling network were identified using the MCODE plug-in within Cytoscape (Bader and Hogue, 2003). Settings below were applied to obtain clusters in the network: degree cutoff = 3, haircut = true, fluff = false, node score cutoff = 0.5, k-core = 3 and max depth = 100.

The nodes of each individual module were tested for functional enrichment based on both Reactome and PANTHER annotations using PANTHER Classification System as described in previous sub-section '**Functional annotation and enrichment analysis'** (Mi and Thomas, 2009, Croft et al., 2011, Mi et al., 2019a).

Enrichment of cell type specific marker genes

Cell type signature gene sets for murine intestinal epithelial cells were obtained from Haber et al. (Haber et al., 2017). Both droplet and plate-based results were used. Gene symbols were converted to Ensembl IDs using db2db (Mudunuri et al., 2009). Hypergeometric significance calculations were applied to test the presence of cell type specific signatures in the list of differentially expressed genes using all expressed genes as the statistical background (normalised counts > 1 in ≥ 1 sample). Bonferroni multiple correction was applied and any corrected $p < 0.05$ was deemed significant. Genes with normalised counts > 1 in ≥ 1 sample per condition (*B. breve* UCC2003 treated or control) were used to identify cell type signature genes expressed per condition.

Key regulator analysis

All mouse transcription factor - target gene interactions with quality scores A-D were obtained from DoRothEA v2 *via* the OmniPath Cytoscape app (Garcia-Alonso et al., 2019, Shannon et al., 2003, Turei et al., 2016). A subnetwork was generated consisting of differentially expressed stem cell signature genes and all their upstream TFs which were expressed in the transcriptomics dataset (normalised counts > 1 in ≥ 1 sample). These TFs were further filtered for their relevance in the network. Here all expressed genes and their upstream expressed TFs were extracted from the DoRothEA network. A hypergeometric significance test was carried out on any node with degree ≥ 5 to determine if the proportion of connected nodes which are differentially expressed is higher than in the whole network. Any TF with $p < 0.05$ following Benjamini-Hochberg correction were deemed significant and used to filter the stem cell signature gene subnetwork. Network visualisation was carried out in Cytoscape (Shannon et al., 2003). Functional enrichment carried out against Reactome pathways as described in previous sub-sections.

Statistical analyses and graphing

Student *t*-tests were performed using Rv.3.6.0, details of which were provided in the results and figure legends (R Development Core Team, 2010). LDA statistical tests for microbiome analysis was performed via LEfSe on Galaxy platform using default parameters (Segata et al., 2011, Jalili et al., 2020). PCA was performed via R library *ggfortify* function *autoplot* and *prcomp*, while Shannon diversity index was computed via R library *vegan* (Dixon, 2003, Tang et al., 2016, R Development Core Team, 2010). All other relevant statistical analyses (including enrichment analysis) were performed within specific software and details were provided in figure legends or as described in the previous sections.

All statistical graphs were either plotted using R library *ggplot2* or *Sleuth* (Wickham, 2016, Pimentel et al., 2017). Heatmaps were graphed using R library *gplots* function *heatmap.2* (Warnes et al., 2016).

Ethics approval
Animal experiments were performed under the UK Regulation of Animals (Scientific Procedures) Act of 1986. The project licence (PPL 80/2545) under which these studies were carried out was approved by the UK Home Office and the UEA Ethical Review Committee. Mice were sacrificed by CO_2 and cervical dislocation.

SUPPLEMENTAL REFERENCES

ALCON-GINER, C., DALBY, M. J., CAIM, S., KETSKEMETY, J., SHAW, A., SIM, K., LAWSON, M., KIU, R., LECLAIRE, C., CHALKLEN, L., KUJAWSKA, M., MITRA, S., FARDUS-REID, F., BELTEKI, G., MCCOLL, K., SWANN, J. R., KROLL, J. S., CLARKE, P. & HALL, L. J. 2019. Microbiota supplementation with Bifidobacterium and Lactobacillus modifies the preterm infant gut microbiota and metabolome. *bioRxiv*, 698092.

BADER, G. D. & HOGUE, C. W. 2003. An automated method for finding molecular complexes in large protein interaction networks. *BMC Bioinformatics,* 4, 2.

BRAY, N. L., PIMENTEL, H., MELSTED, P. & PACHTER, L. 2016. Near-optimal probabilistic RNA-seq quantification. *Nat Biotechnol,* 34, 525-7.

BULT, C. J., BLAKE, J. A., SMITH, C. L., KADIN, J. A., RICHARDSON, J. E. & MOUSE GENOME DATABASE, G. 2019. Mouse Genome Database (MGD) 2019. *Nucleic Acids Res,* 47, D801-D806.

CAPORASO, J. G., KUCZYNSKI, J., STOMBAUGH, J., BITTINGER, K., BUSHMAN, F. D., COSTELLO, E. K., FIERER, N., PENA, A. G., GOODRICH, J. K., GORDON, J. I., HUTTLEY, G. A., KELLEY, S. T., KNIGHTS, D., KOENIG, J. E., LEY, R. E., LOZUPONE, C. A., MCDONALD, D., MUEGGE, B. D., PIRRUNG, M., REEDER, J., SEVINSKY, J. R., TURNBAUGH, P. J., WALTERS, W. A., WIDMANN, J., YATSUNENKO, T., ZANEVELD, J. & KNIGHT, R. 2010. QIIME allows analysis of high-throughput community sequencing data. *Nat Methods,* 7, 335-6.

CHEN, S., ZHOU, Y., CHEN, Y. & GU, J. 2018. fastp: an ultra-fast all-in-one FASTQ preprocessor. *Bioinformatics,* 34, i884-i890.

CROFT, D., O'KELLY, G., WU, G., HAW, R., GILLESPIE, M., MATTHEWS, L., CAUDY, M., GARAPATI, P., GOPINATH, G., JASSAL, B., JUPE, S., KALATSKAYA, I., MAHAJAN, S., MAY, B., NDEGWA, N., SCHMIDT, E., SHAMOVSKY, V., YUNG, C., BIRNEY, E., HERMJAKOB, H., D'EUSTACHIO, P. & STEIN, L. 2011. Reactome: a database of reactions, pathways and biological processes. *Nucleic Acids Res,* 39, D691-7.

CUNNINGHAM, F., ACHUTHAN, P., AKANNI, W., ALLEN, J., AMODE, M. R., ARMEAN, I. M., BENNETT, R., BHAI, J., BILLIS, K., BODDU, S., CUMMINS, C., DAVIDSON, C., DODIYA, K. J., GALL, A., GIRON, C. G., GIL, L., GREGO, T., HAGGERTY, L., HASKELL, E., HOURLIER, T., IZUOGU, O. G., JANACEK, S. H., JUETTEMANN, T., KAY, M., LAIRD, M. R., LAVIDAS, I., LIU, Z., LOVELAND, J. E., MARUGAN, J. C., MAUREL, T., MCMAHON, A. C., MOORE, B., MORALES, J., MUDGE, J. M., NUHN, M., OGEH, D., PARKER, A., PARTON, A., PATRICIO, M., ABDUL SALAM, A. I., SCHMITT, B. M., SCHUILENBURG, H., SHEPPARD, D., SPARROW, H., STAPLETON, E., SZUBA, M., TAYLOR, K., THREADGOLD, G., THORMANN, A., VULLO, A., WALTS, B., WINTERBOTTOM, A., ZADISSA, A., CHAKIACHVILI, M., FRANKISH, A., HUNT, S. E., KOSTADIMA, M., LANGRIDGE, N., MARTIN, F. J., MUFFATO, M., PERRY, E., RUFFIER, M., STAINES, D. M., TREVANION, S. J., AKEN, B. L., YATES, A. D., ZERBINO, D. R. & FLICEK, P. 2019. Ensembl 2019. *Nucleic Acids Res,* 47, D745-D751.

DIXON, P. 2003. VEGAN, a package of R functions for community ecology. *J Veg Sci,* 14, 927-930.

HUSON, D. H., BEIER, S., FLADE, I., GORSKA, A., EL-HADIDI, M., MITRA, S., RUSCHEWEYH, H. J. & TAPPU, R. 2016. MEGAN Community Edition - Interactive Exploration and Analysis of Large-Scale Microbiome Sequencing Data. *PLoS Comput Biol,* 12, e1004957.

JALILI, V., AFGAN, E., GU, Q., CLEMENTS, D., BLANKENBERG, D., GOECKS, J., TAYLOR, J. & NEKRUTENKO, A. 2020. The Galaxy platform for accessible, reproducible and collaborative biomedical analyses: 2020 update. *Nucleic Acids Res.*

KINSELLA, R. J., KAHARI, A., HAIDER, S., ZAMORA, J., PROCTOR, G., SPUDICH, G., ALMEIDA-KING, J., STAINES, D., DERWENT, P., KERHORNOU, A., KERSEY, P. & FLICEK, P. 2011. Ensembl BioMarts: a hub for data retrieval across taxonomic space. *Database (Oxford),* 2011, bar030.

KIU, R., BROWN, J., BEDWELL, H., LECLAIRE, C., CAIM, S., PICKARD, D., DOUGAN, G., DIXON, R. A. & HALL, L. J. 2019. Genomic analysis on broiler-associated Clostridium perfringens strains and exploratory caecal microbiome investigation reveals key factors linked to poultry necrotic enteritis. *Animal Microbiome,* 1, 12.

KOPYLOVA, E., NOE, L. & TOUZET, H. 2012. SortMeRNA: fast and accurate filtering of ribosomal RNAs in metatranscriptomic data. *Bioinformatics,* 28, 3211-7.

MI, H., MURUGANUJAN, A., EBERT, D., HUANG, X. & THOMAS, P. D. 2019a. PANTHER version 14: more genomes, a new PANTHER GO-slim and improvements in enrichment analysis tools. *Nucleic Acids Res,* 47, D419-D426.

MI, H., MURUGANUJAN, A., HUANG, X., EBERT, D., MILLS, C., GUO, X. & THOMAS, P. D. 2019b. Protocol Update for large-scale genome and gene function analysis with the PANTHER classification system (v.14.0). *Nat Protoc,* 14, 703-721.

MI, H. & THOMAS, P. 2009. PANTHER pathway: an ontology-based pathway database coupled with data analysis tools. *Methods Mol Biol,* 563, 123-40.

MUDUNURI, U., CHE, A., YI, M. & STEPHENS, R. M. 2009. bioDBnet: the biological database network. *Bioinformatics,* 25, 555-6.

PIMENTEL, H., BRAY, N. L., PUENTE, S., MELSTED, P. & PACHTER, L. 2017. Differential analysis of RNA-seq incorporating quantification uncertainty. *Nat Methods,* 14, 687-690.

QUAST, C., PRUESSE, E., YILMAZ, P., GERKEN, J., SCHWEER, T., YARZA, P., PEPLIES, J. & GLOCKNER, F. O. 2013. The SILVA ribosomal RNA gene database project: improved data processing and web-based tools. *Nucleic Acids Res,* 41, D590-6.

R DEVELOPMENT CORE TEAM 2010. R: A language and environment for statistical computing. *R Foundation for Statistical Computing.* Vienna, Austria.

SEGATA, N., IZARD, J., WALDRON, L., GEVERS, D., MIROPOLSKY, L., GARRETT, W. S. & HUTTENHOWER, C. 2011. Metagenomic biomarker discovery and explanation. *Genome Biol,* 12, R60.

SHANNON, P., MARKIEL, A., OZIER, O., BALIGA, N. S., WANG, J. T., RAMAGE, D., AMIN, N., SCHWIKOWSKI, B. & IDEKER, T. 2003. Cytoscape: a software environment for integrated models of biomolecular interaction networks. *Genome Res,* 13, 2498-504.

TANG, Y., HORIKOSHI, M. & LI, W. X. 2016. ggfortify: Unified Interface to Visualize Statistical Result of Popular R Packages. *The R Journal,* 8.

TUREI, D., KORCSMAROS, T. & SAEZ-RODRIGUEZ, J. 2016. OmniPath: guidelines and gateway for literature-curated signaling pathway resources. *Nat Methods,* 13, 966-967.

WARNES, G. R., BOLKER, B., BONEBAKKER, L., GENTLEMAN, R., HUBER, W., LIAW, A., LUMLEY, T., MAECHLER, M., MAGNUSSON, A., MOELLER, S., SCHWARTZ, M. & VENABLES, B. 2016. gplots: Various R Programming Tools for Plotting Data. R package version 3.0.1 ed.

WICKHAM, H. 2016. *ggplot2: Elegant Graphics for Data Analysis,* New York, Springer-Verlag.

ZERBINO, D. R., ACHUTHAN, P., AKANNI, W., AMODE, M. R., BARRELL, D., BHAI, J., BILLIS, K., CUMMINS, C., GALL, A., GIRON, C. G., GIL, L., GORDON, L., HAGGERTY, L., HASKELL, E., HOURLIER, T., IZUOGU, O. G., JANACEK, S. H., JUETTEMANN, T., TO, J. K., LAIRD, M. R., LAVIDAS, I., LIU, Z., LOVELAND, J. E., MAUREL, T., MCLAREN, W., MOORE, B., MUDGE, J., MURPHY, D. N., NEWMAN, V., NUHN, M., OGEH, D., ONG, C. K., PARKER, A., PATRICIO, M., RIAT, H. S., SCHUILENBURG, H., SHEPPARD, D., SPARROW, H., TAYLOR, K., THORMANN, A., VULLO, A., WALTS, B., ZADISSA, A., FRANKISH, A., HUNT, S. E., KOSTADIMA, M., LANGRIDGE, N., MARTIN, F. J., MUFFATO, M., PERRY, E., RUFFIER, M., STAINES, D. M., TREVANION, S. J., AKEN, B. L., CUNNINGHAM, F., YATES, A. & FLICEK, P. 2018. Ensembl 2018. *Nucleic Acids Res,* 46, D754-D761.
